# Light management in monolithic all-perovskite tandem solar cells

**DOI:** 10.1038/s41377-025-02120-5

**Published:** 2026-01-04

**Authors:** Chenshuaiyu Liu, Han Gao, Wennan Ou, Hairen Tan, Renxing Lin

**Affiliations:** National Laboratory of Solid State Microstructures, College of Engineering and Applied Sciences, Frontiers Science Center for Critical Earth Material Cycling, Nanjing University, Nanjing, 210023 China

**Keywords:** Photonic devices, Optical materials and structures

## Abstract

All-perovskite tandem solar cells represent a promising strategy for breaking the Shockley-Queisser limits inherent in single-junction solar cells. Reasonable light management and optical design are necessary for all-perovskite tandem solar cells to improve power conversion efficiency. In this review, the recent progresses in light management for monolithic all-perovskite tandem solar cells are summarized comprehensively. The current-matching conditions, optical challenges, and potential development trajectories for all-perovskite tandem solar cells are investigated. It includes key optical losses, enhancements and strategies for light trapping and light utilization. Ultimately, forward-looking perspectives on future developments are presented. This review aims to offer valuable insights and practical suggestions for improving power conversion efficiency of all-perovskite tandem solar cells from light management techniques.

## Abstract (web summary)

Summary diagram of light management in monolithic all-perovskite tandem solar cells.

## Graphical abstract

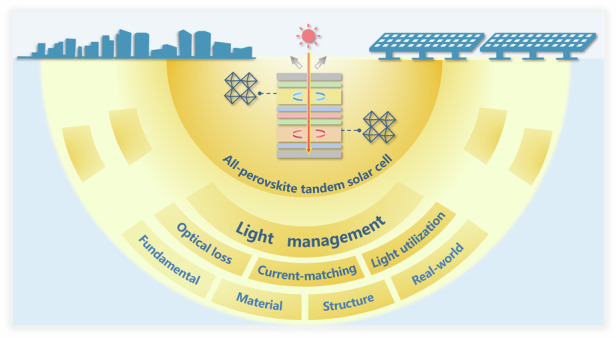

## Introduction

All-perovskite tandem solar cells (APTSCs), comprised of wide-bandgap (WBG, *E*_g_ ~ 1.8 eV) and narrow-bandgap (NBG, *E*_g_ ~ 1.2 eV) perovskite sub-cells, have achieved higher power conversion efficiency (PCE) compared to single-junction perovskite solar cells (PSCs) in a short period of development^1–9^. The best performance two-terminal (2 T) APTSCs so far have demonstrated a PCE of 30.1%, with the open-circuit voltage (*V*_oc_) of 2.20 V, short-circuit current density (*J*_sc_) of 16.7 mA cm^–2^ and fill factor (FF) of 81.8%^10^. However, the *J*_sc_ of state-of-the-art double-junction APTSCs (2J-APTSCs) is limited to 16.7 mA cm^–2^, which is significantly below the theoretical predicted value (~18.0 mA cm^–^^2^)^11^. A key factor contributing to this discrepancy is the underutilization of light, which presents a pivotal challenge that constrains the PCE of tandem devices.

**Fig. 1:**
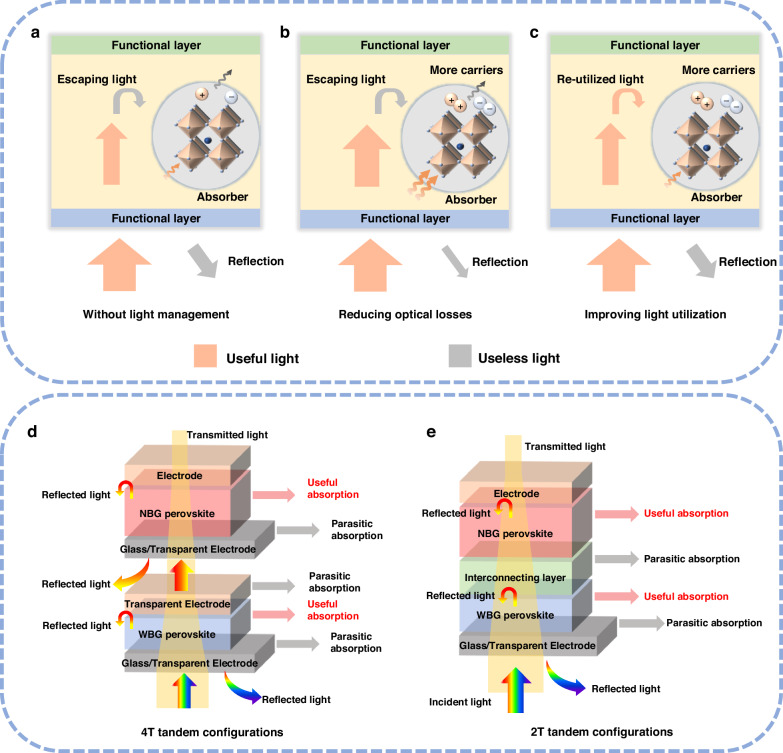
The schematic diagram of light management and APTSCs. **a** PSCs without light management, resulting photons either not entering the absorber layer nor escaping from the absorber layer. **b** Reducing optical losses to maximize photon flux reaching the absorber. **c** Improving light utilization to ensure photon captured by the absorber and efficiently converted into charge carriers. **d** 4 T tandem configurations. **e** 2 T tandem configurations

Light management exerts a significant impact on photocurrent generation in APTSCs^12,13^. Significant optical losses originate from interfacial reflection and parasitic absorption within the functional layers, occurring before photons reach the perovskite absorber. Additional losses occur within the absorber itself, originating from not only insufficient photon absorption but also parasitic carrier transport (Fig. 1a). Consequently, light management strategies can be categorized into two complementary approaches based on the pathway of photons. The first focuses on minimizing external optical losses to maximize the number of photons entering the absorber (Fig. 1b). The second is enhancing the photon capture ability of absorbers and improving the photon-to-carrier conversion efficiency, thereby producing more electron–hole pairs (Fig. 1c). Single-junction PSCs can easily achieve high *J*_sc_ by simply reducing the parasitic absorption of the inactive layer and thickening the active layer to increase the harvesting of photons. Compared to single-junction PSCs, APTSCs with the multilayer films contributes significantly to parasitic absorption and reflection losses (Fig. 1d, e). The design of more layers and the introduction of the interconnecting layers (ICLs) not only increases optical losses but also imposes additional constraints on spectral allocation and current matching between sub-cells (Fig. 1e). In addition, APTSCs face an additional critical conflict in the harvesting of near-infrared (NIR) photons: the inherently low absorption coefficients for NIR light and high defect densities of mixed Pb-Sn NBG perovskites. Concurrently, thin-film interference poses a new challenge to optimal current matching in the back sub-cell. These factors create a critical trade‑off not present in single‑junction PSCs, and thus necessitate dedicated light‑management tailored to APTSC architectures.

In this review, we summarize recent advancements in light management strategies for APTSCs and propose suggestions for future optimization, with the anticipation of further improving the PCE of APTSCs. Section “Optical design principle for APTSCs” discusses the structure of APTSCs and presents a reference optical design principle. In Section “Optical losses of APTSCs” and Section “Improving light utilization”, the optical losses, light capture and light utilization of APTSCs are explored. The issues and challenges encountered by each functional layer are analyzed, and effective strategies are summarized. In Section “Conclusion and outlook”, we outline the future prospect for APTSCs, aiming to provide a guidance for achieving photovoltaic (PV) performance breakthrough in the field.

## Optical design principle for APTSCs

APTSCs can be divided into four-terminal (4 T) and two-terminal (2 T) tandem configurations according to the structure. The two sub-cells are prepared separately and mechanically stacked in 4 T tandem devices (shown in Fig. 1d). The two sub-cells work independently without current-matching condition. The reduction of optical losses (from parasitic absorption, reflection and so on) is of paramount importance in 4 T tandem solar cells. To achieve optimal performance, the front WBG sub-cells require high optical transmission in their charge transport layers (CTL) and electrodes. The three electrodes positioned along the light’s path—the front sub-cell’s two electrodes and the back sub-cell’s front electrode—should ideally act as transparent electrodes. Considering NIR transparency is essential, since the absorption portion of the back sub-cells is concentrated in this region. However, for many transparent conductive oxides, parasitic absorption in the NIR region can be a significant loss channel, reducing the photocurrent of the back sub-cells. Furthermore, precisely tuning the bandgap of the WBG sub-cells enables the implementation of an optimal spectral splitting design, which is essential for maximizing power output in tandem solar cells^14^.

Distinct from 4 T tandem solar cells, 2 T tandem configurations exhibited a unified all-perovskite architecture in which the WBG and NBG sub-cells are directly connected in series through an ICL (as shown in Fig. 1e). Due to the direct serial connection between the two sub-cells, according to Kirchhoff’s Current Law, the current in the series tandem cell is constrained by the sub-cell with the smaller one. Consequently, precise optical matching of the two sub-cells is essential in 2 T designs to ensure balanced current generation and to maximize the overall current output. Furthermore, the ICL in the 2 T tandem cells must be robust enough. It should tolerate exposure to solvents (such as DMSO, DMF, etc.) used during the fabrication of the bottom sub-cell on the top sub-cell. Additionally, the ICLs must exhibit minimal optical parasitic absorption to avoid incident losses before NBG sub-cell. Lastly, the energy level of ICL needs to align with both sub-cells to achieve optimal electrical series connection, thereby mitigating electrical losses.

When comparing tandem configurations, the 4 T configuration offers a fabrication process that is relatively simpler than the 2 T tandem. However, it is accompanied by significant optical losses, which are attributed not only to parasitic absorption by the three electrodes, but also to additional losses from parasitic absorption and reflections resulting from the increased number of interfaces. Conversely, despite the greater complexity required for the 2 T configuration, it exhibits lower optical losses and is capable of achieving better current matching. Unless otherwise specified, this review will concentrate solely on the optical design and light management aspects of 2 T tandem configurations. As for the photocurrent matching condition, the bandgap and thickness of the perovskite absorber layer needs to be emphasized for 2 T APTSCs. The essence of current-matching is the distribution of solar spectrum and photons by the two sub-cells. Sunlight is incident from the transparent electrode side and first absorbed by the WBG sub-cell, whose properties determine the range and number of photons allowed to pass through. The remaining photons are absorbed by the NBG sub-cell. It is pivotal to explore the relationship between the bandgap and thickness of two sub-cells to meet the matching-photocurrent of APTSCs.

### Bandgap engineering for spectrum allocation

**Fig. 2:**
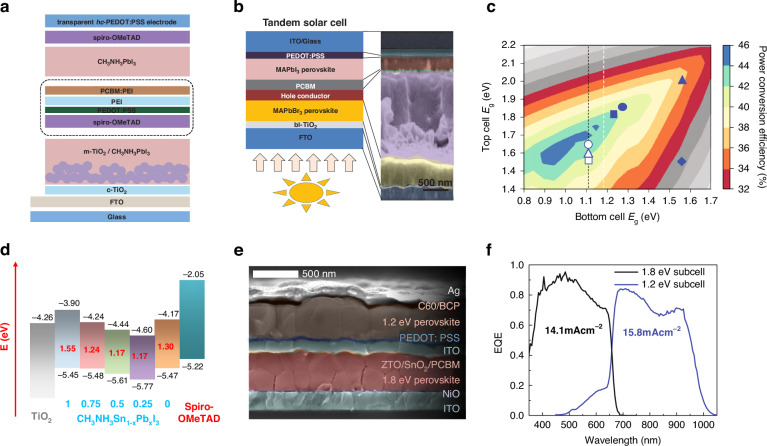
Bandgaps design for the APTSCs. **a** The schematic diagram of APTSCs constructed by two MAPbI_3_ perovskite layers. **b** The schematic diagram of APTSCs constructed by MAPbBr_3_ and MAPbI_3_ perovskite. **c** Theoretical maximum PCEs as a function of the top and back sub-cell bandgaps for 2 T tandem configurations. The white dotted lines mark the lowest bandgap currently accessible to metal halide perovskite semiconductors. The blue solid symbols show bandgap combinations thus far used in making all-perovskite tandems, including 1.82/1.22 eV, 1.85/1.27 eV, 2.0/1.55 eV and 1.55/1.55 eV. **d** The bowing effect, which is the schematic energy level diagram of the CH_3_NH_3_Sn_1−x_Pb_x_I_3_ (MASn_1−x_Pb_x_I_3_) NBG perovskites. **e** Scanning electron microscope (SEM) image of APTSCs constructed by Pb-Sn perovskite as the back sub-cell. **f** EQE response values of sub-cells of Fig. 2e. Panel **a** is reprinted from ref. ^15^ with permission from Royal Society Chemistry. Panel **b** is reprinted from ref. ^18^ with permission from Wiley. Panel **c** is reprinted from ref. ^19^ with permission from Springer Nature. Panel **d** is reprinted from ref. ^26^ with permission from American Chemical Society. Panels **e** and **f** are reprinted from ref. ^27^ with permission from American Chemical Society

In the early development of APTSCs, the two sub-cells were composed of identical perovskite materials. Jiang et al. proposed an APTSCs with two CH_3_NH_3_PbI_3_ (MAPbI_3_) layers connected by ICL structure (Fig. 2a)^15^. This APTSC employs a perovskite film as the front absorber layer to permit controlled transmission of NIR photons for absorption by the back sub-cell. However, the non-complementary spectra between the two sub-cells and the mesoporous layer with scattering characteristics make this APTSC demonstrated a quite unbalanced *J*_sc_ (a *J*_sc_ of 6.61 mA cm^–2^ and a PCE of ~7.0%). In fact, the flexibility of perovskite composition makes the bandgap of perovskite materials adjustable at 1.2–3.0 eV^16,17^. Heo et al. used 2.2 eV MAPbBr_3_ perovskite as the front sub-cell of APTSCs via a complete substitution with I^−^by Br^−^ (Fig. 2b)^18^. The optimized performance was also unsatisfactory, ~10.8%. This suggested that the efforts on perovskites with larger bandgaps serried in 2J-APTSCs were invalid because the longer-wavelength light in the solar spectrum was not utilized.

In pursuit of making full use of the solar spectrum and maximizing the photocurrent in the two-junction cells, the bandgaps of the two perovskite sub-cells need to be regulated. Leijtens et al. depicted the relationship between theoretical PCE and bandgaps of double-junction tandem solar cells, which is shown in Fig. 2c^19^. To achieve optimal photoelectronic conversion efficiency in double-junction tandem solar cells, the theoretically ideal NBG and WBG configurations should be maintained at 1.0–1.1 eV and 1.5–1.7 eV, respectively. Cu(In, Ga)Se_2_ solar cells can readily be tuned to this target through compositional engineering, whereas silicon cells inherently possess a fixed bandgap (around 1.1 eV) that fortuitously aligns with this requirement^20–25^. As for APTSCs, NBG perovskite can be realized by introducing Sn at the B-site (shown in Fig. 2d)^26^. However, state-of-the-art NBG perovskite sub-cells currently operate at around 1.22 eV, approaching the material’s intrinsic minimum bandgap (1.2 eV), thereby leaving little room for further bandgap reduction. Given the inherent bandgap limitations of NBG perovskite sub-cells, optimizing WBG perovskite bandgap becomes a key priority to achieve effective current matching in APTSCs. It is noteworthy that the bandgap of mixed Pb-Sn perovskite does not change linearly with composition beyond 50% Sn content, which is referred to as the bowing effect^26^. When Sn content is 50–60%, the mixed Pb-Sn perovskite exhibits the narrowest bandgap. Eperon and co-workers first proposed to construct APTSCs by Pb-Sn perovskite to capture NIR light, and the architecture is shown in Fig. 2e^27^. The range of absorbable spectrum was broadened to 1100 nm, demonstrating the operability of Pb-Sn perovskite as the back sub-cell. Unfortunately, serious photocurrent loss occurred, the photocurrent of the 1.2 eV sub-cell was much larger than that of the 1.8 eV sub-cell (Fig. 2f). The PCE of the device was only 16.9%, with the *J*_sc_ of 14.5 mA cm^–2^. This study shows the potential of APTSCs to achieve higher PCE from the perspective of bandgap matching. The mixed Pb-Sn perovskites play an important role in the construction of APTSCs and absorption of NIR light, and have become popular materials for research on APTSCs.

### Thickness adjustment for photon allocation

In addition to bandgap engineering, optimizing the thickness of the absorbers is equally essential for effective photon management. As multilayer thin-film optical systems, APTSCs rely heavily on the optical properties, layer thicknesses, and stacking sequence of their constituent layers^28^. In APTSCs, which incorporate two light-absorbing layers, the optical design becomes more complex than in single-junction PSCs due to the need for balanced photon absorption and current matching between sub-cells.

**Fig. 3:**
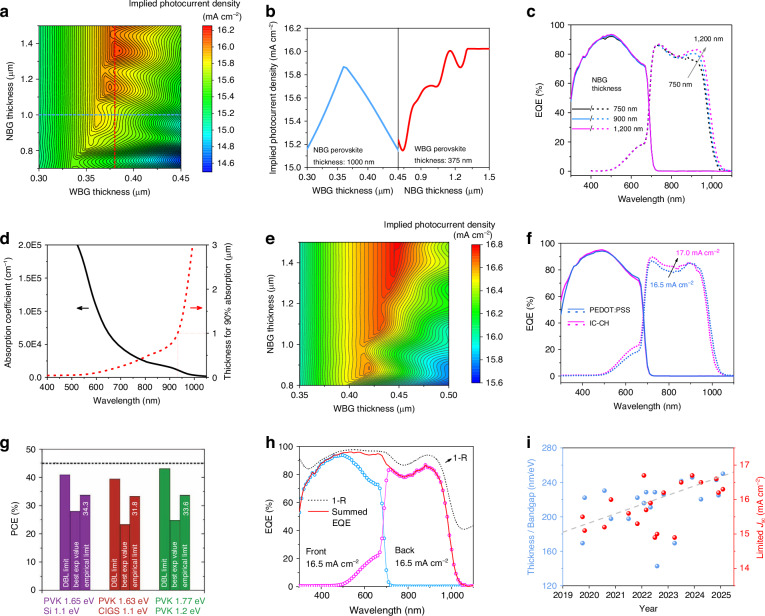
Thickness adjustment for the APTSCs. **a** Implied photocurrent density as a function of the front and back sub-cell thickness for APTSC with Au-based ICL. The implied current density shown by the dashed line is shown in (**b**)**. c** EQE response values of APTSCs with different NBG perovskite thicknesses. **d** Absorption coefficient of FA_0.5_MA_0.45_Cs_0.05_Pb_0.5_Sn_0.5_I_3_ (with a bandgap of 1.22 eV), and corresponded thickness of the films. **e** Implied photocurrent density as a function of the front and back sub-cell thickness for APTSC with ITO NCs-based ICL. **f** EQE curves of APTSCs with Au-based and ITO NCs-based ICL. **g** Detailed balance limit (DBL) for series-connected tandem solar cells limit based on the bandgap combination. **h** EQE and total reflection (1-R) curves of state-of-the-art APTSCs. **i** Development of WBG perovskite properties and the limited *J*_sc_ of the corresponding APTSCs. The properties of WBG perovskite are defined by the ratio of thickness to bandgap without specific physical meaning, and the limited *J*_sc_ depends on a sub-cell with a smaller *J*_sc_, derived from the EQE curves. Panels **a**, **c**, and **h** are reprinted from ref. ^29^ with permission from Springer Nature. Panel **d** is reprinted from ref. ^31^ with permission from Springer Nature. Panels **e** and **f** are reprinted from ref. ^33^ with permission from Wiley. Panel **g** is reprinted from ref. ^11^ with permission from Wiley

Due to the interplay between the two sub-cells in APTSCs, a critical aspect of optimizing PV performance lies in the precise regulation of the thickness of the two absorbers. Lin et al. made an in-depth study on the thickness control for APTSCs^29^. The typical APTSCs device structure was utilized as the model, with NiO_x_ serving as the hole transport layer for WBG sub-cell, and C_60_/SnO_2_/Au/PEDOT: PSS acting as the ICL. The implied photocurrent density was calculated as a function of the thickness of WBG and NBG absorber layers (as shown in Fig. 3a). It showed the thickness of both two absorbers play a significant role in the photocurrent of APTSCs. The front WBG perovskite acts as the incident side, with its thickness determining the quantity of photons that can pass through. Thick WBG perovskite film would absorb almost all the photons within its bandgap (<750 nm), thus NBG perovskite could not achieve the photocurrent matching with the WBG sub-cell only by the remaining NIR photons. According to Kirchhoff’s current law, the photocurrent of APTSCs would primarily depend on the NBG sub-cell due to its lower photocurrent. However, if the WBG perovskite film is excessively thin, the photocurrent of the APTSCs will instead be dictated by the WBG sub-cell. In this context, it is essential to carefully control the thickness of both the front WBG and the back NBG absorbers to achieve current-matching, thereby pursuing the optimal photoelectric conversion efficiency. Once the thickness of NBG perovskite was constant (e.g., 1000 nm), WBG perovskite should be adjusted to a suitable thickness to maximize the photogenerated current of APTSCs (blue line in Fig. 3b, left panel).

Regarding the NBG perovskite, it is advisable to maximize its thickness to ensure effective absorption of NIR photons (Fig. 3c)^30^. Under the precise control of WBG thickness, the photocurrent of APTSCs would enhance with the increase of NBG perovskite thickness in general (red line in Fig. 3b, right panel). Yang et al. also investigated the absorption properties of FA_0.5_MA_0.45_Cs_0.05_Pb_0.5_Sn_0.5_I_3_ with the bandgap of 1.22 eV^31^. They found that the thickness of the NBG perovskite films should reach at least 1 μm to exhibit satisfactory absorption in the NIR region (Fig. 3d). However, it should be noted that the photocurrent does not monotonically increase with the thickness of NBG perovskite, it may decrease at certain thickness range instead. For instance, when the WBG perovskite is 375-nm-thick and the NBG perovskite ranges between 1.1 and 1.2 μm, a decline in photocurrent is observed (red line in Fig. 3b, right panel). This can be attributed to interference effects. The whole APTSCs can be regarded as a Fabry-Pérot (F-P) resonator. The standing waves generated by multiple beams of incident lights and reflected lights are coupled with excitons generated by the absorbers, that is, Fano resonance^32^. Thus, the intensity of light does not change linearly with the absorber thickness, and the enhancement of the thickness of NBG film should also consider the thickness of WBG perovskite to ensure the constructive interference.

In addition to the thickness of the absorbers, the optical properties of the functional layer will bring about a great impact on the photon balance. Liu et al. tried to replace Au/PEDOT: PSS ICL with the optically better structure of tin-doped indium oxide nanocrystals (ITO NCs)/self-assembled monolayers (SAMs), the model results were completely different in both the implied photocurrent value and the corresponding perovskite thickness, which is shown in Fig. 3e^33^. The enhancement on optical properties would be manifested in the benefit of the photocurrent of APTSCs, and also significantly affect the shape of the absorption curve of the back NBG sub-cell (shown in Fig. 3f). Changes in optical properties would inevitably destroy the original photon balance, thus the bandgap and thickness of the two absorbers should be adjusted to maintain the current-matching conditions and minimize the photocurrent losses.

### Challenge of optical design for APTSCs

The optical simulations based on the detailed balance limit (DBL) demonstrated that 2J-APTSCs could achieve a PCE exceeding 40% with a *J*_sc_ approaching 20 mA cm^-2^ under ideal conditions (Fig. 3g)^11^. This theoretical model assumes negligible external quantum loss, where each incident photon generates an electron-hole pair through complete conversion within the perovskite absorber. In such idealized scenarios, sub-cells could achieve maximized current matching through equal photon distribution. However, various loss paths in practical devices lead to a mismatch in the photocurrents generated for the two sub-cells, resulting in a significant deviation from theoretical predictions. The spectral dependence of the external quantum efficiency stems from optical and recombination losses tied to device structure and material properties. Therefore, the photocurrents in the two sub-cells differ even under spectrally balanced illumination^34^. In this case, the independence of each sub-cell becomes crucial. The light management of APTSCs should always consider minimizing the current-gap and avoiding Fabry-Pérot interference effects between the two sub-cells during individual optimization.

In 2J-APTSCs, optical losses of WBG sub-cell are primarily attributed to glass/air interface reflections and TCO electrode parasitic absorption. Although advanced high-transparency TCOs, optimized HTLs, and anti-reflective coatings can further minimize optical losses, the summed EQE below 600 nm is remarkably close to the theoretical optical limit (1-R, Fig. 3h)^35^, meaning that the remaining potential for further WBG photocurrent enhancement through optical engineering has become substantially constrained. In contrast, NBG sub-cells experience significant parasitic absorption and reflection losses from the glass/air interface, front TCO electrode, ICL, and HTL. Optimizing the HTL and ICL to reduce parasitic absorption, along with improving NIR photon harvesting, can significantly enhance the photocurrent in NBG sub-cells. Although light management can increase the *J*_sc_ in NBG sub-cell, it breaks the original current balance. To achieve a new match and enhance a comprehensive performance in tandem, a re-optimization for the two sub-cells is essential, including the bandgap and thickness accordingly.

Specifically, the WBG absorber thickness needs to be precisely adjusted, so that it can achieve enhanced interference effects for the optimal NBG sub-cell, thereby maximizing its photocurrent generation. However, such optimized interference configuration sometimes does not ensure current matching between the two sub-cells. So, the additional bandgap engineering for the WBG perovskite layer under maintained absorber thickness is necessary. We summarize the thickness and bandgap of the WBG sub-cell in APTSCs in Table 1 and Fig. 3i. The results reveal a clear trend emerges in the WBG sub-cells development following the systematic bandgap narrowing and active layer thickening. This trend is aimed at the improved spectral utilization while sustaining current matching with the enhanced *J*_sc_ of NBG PSCs. Notably, NBG sub-cells also demonstrate the same evolving trends. These distinct lines of development are systematically examined in Section “Transparent charge transport layer and interconnecting layer” (ICL engineering) and 4.1 (NBG perovskite advancements).

**Table 1:** Thickness and bandgap of WBG perovskite, and the limited *J*_sc_ of APTSCs

Year	Thickness (nm)	Bandgap (eV)	Thickness/Bandgap (nm/eV)	Limited *J*_sc_ (mA cm^–2^)	Ref.
2019	300	1.77	169.5	15.5	Ref. ^83^
2019	400	1.80	222.2	15.1	Ref. ^31^
2020	410	1.78	230.3	15.2	Ref. ^86^
2020	350	1.77	197.7	16.0	Ref. ^96^
2021	350	1.77	197.7	15.6	Ref. ^146^
2021	400	1.80	222.2	15.3	Ref. ^84^
2022	380	1.76	215.9	16.7	Ref. ^29^
2022	400	1.75	228.6	15.7	Ref. ^147^
2022	380	1.80	211.1	15.9	Ref. ^148^
2022	400	1.75	228.6	14.9	Ref. ^149^
2022	250	1.75	142.9	15.0	Ref. ^79^
2022	400	1.77	226.0	16.2	Ref. ^85^
2023	300	1.77	169.5	14.9	Ref. ^82^
2023	430	1.78	241.6	16.5	Ref. ^35^
2023	430	1.75	245.7	16.7	Ref. ^33^
2024	390	1.77	220.3	16.5	Ref. ^150^
2024	430	1.77	242.9	16.6	Ref. ^151^
2024	405	1.80	225.0	16.2	Ref. ^152^
2025	450	1.80	250.0	16.3	Ref. ^153^

Thickness was obtained from SEM images, bandgap was obtained from methods, and the limited *J*_sc_ was referred to the sub-cell with smaller *J*_sc_ and was obtained from EQE measurement

Achieving precise current matching, optimizing the thickness and bandgap of WBG perovskite, and maximizing the light collection efficiency of NBG sub-cells highly depend on the comprehensive understanding of intricate light field distributions and the photon-to-carrier conversion process. This typically necessitates accurate optical modeling and simulation prediction, as well the analysis and optimization of the device structures. Commonly employed models include ray tracing^36^, transmission matrix (TMM) method^37,38^, finite-difference time-domain (FDTD) method^39,40^, finite element (FEM) method^41^, rigorous coupled-wave analysis (RCWA)^42^ and so on. The applicability of ray tracing optics is constrained to the micro-scale devices where the structure size are hundreds of times larger than the light wavelength. Compared to APTSCs with nano-scale structures, ray tracing optics is more suitable for Si/perovskite tandem solar cells because of prominent textured surface from Si sub-cells. The TMM method is widely utilized for transmission, reflection, EQE spectra and thickness optimization, as in Fig. 3a, e. It facilitates macroscopic light management within devices, precise thickness matching, spectral splitting, and materials optical property design. Nevertheless, TMM simplifies models into one-dimensional (1D) configurations, thus limiting its for nanoparticle stacking, complex textured surfaces, or laterally non-uniform structures. For those 2D or even 3D light-trapping structures, FDTD, FEM, and RCWA offer broader applicability. The FDTD method is able to discretize space and time fields, accurately representing interactions with any nanostructure, including scattering, resonance, and near-field enhancement. The FEM method excels in coupling multiple physical fields and fitting non-uniform materials, thereby enabling effectively electrical simulations of carrier dynamics and thermal simulations of heat transfer. While RCWA surpasses FDTD and FEM in dealing with periodic nanostructures, such as periodic photonic crystals and the grating design of the top electrode. Each simulation method possesses unique advantages and can be synergistically combined in practical applications to ensure APTSCs achieve accurate current matching and minimize optical losses^43^.

In APTSCs, light management strategies fundamentally differ from those employed in single-junction PSCs, due to the combined influence of current matching, sub-cell independence, and complex multilayer interference effects. This paradigm shift necessitates a systematic categorization of light management and an in-depth analysis of their operational mechanisms, as discussed in Section “Introduction”. Critical considerations include the location of these strategies within the device structure, effects on spectral response and photon utilization, and the interactions with other components of the solar cell. Ultimately, this understanding will facilitate the optimization of current matching and enhancement of optoelectronic performance in APTSCs by fine-tuning the bandgap and thickness of the two absorbers.

## Optical losses of APTSCs

**Fig. 4:**
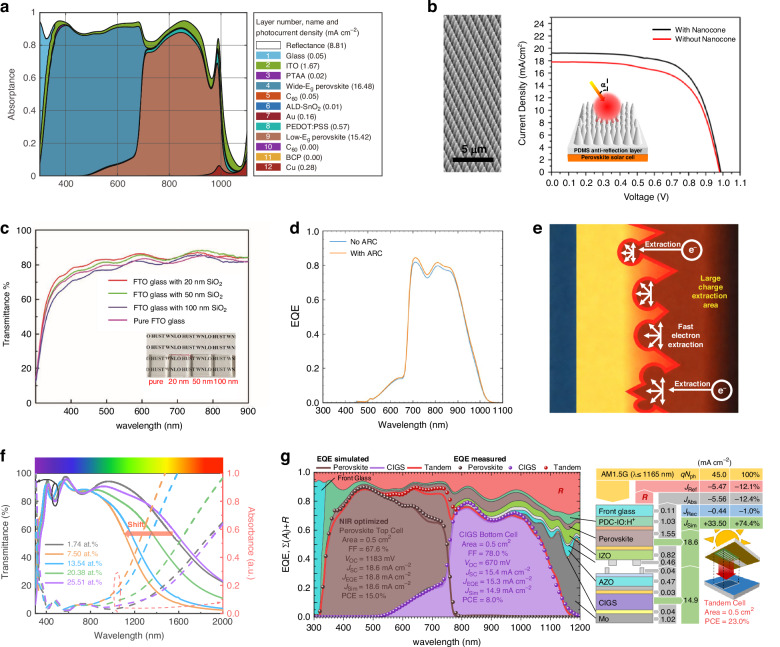
The optical loss by the front reflection and the front electrodes. **a** Simulated spectral absorptance and reflectance of APTSCs. **b** SEM image of PDMS nanocone (left) and *J*-*V* curves of PSCs with and without PDMS nanocone film (right). **c** Transmittance curves of FTO glass without and with SiO_2_ mesoporous films. **d** EQE curve of the NBG sub-cell with and without an anti-reflective coating of MgF_2_ on the glass substrate facing the light. **e** Illustration of the extraction behavior of photoexcited electrons from perovskite into the etched FTO. **f** Transmittance and absorbance spectra of the CE-ITO substrates with different doped-concentration. **g** Spectral evaluation of the EQE (measured and simulated), reflection losses (measured), and absorption losses (simulated) of the tandem cell architecture with a NIR-optimized PDC-IO:H as a front electrode. Panel **a** is reprinted from ref. ^28^ with permission from Elsevier. Panel **b** is reprinted from ref. ^49^ with permission from American Chemical Society. Panel **c** is reprinted from ref. ^54^ with permission from Wiley. Panel **d** is reprinted from ref. ^57^ with permission from Royal Society Chemistry. Panel **e** is reprinted from ref. ^60^ with permission from Wiley. Panel **f** is reprinted from ref. ^61^ with permission from Wiley. Panel **g** is reprinted from ref. ^62^ with permission from American Chemical Society

Although careful bandgap matching and thickness tuning ensure that the sub-cells can absorb the right portion of the solar spectrum, this does not guarantee that all incident photons actually reach those absorbers. A critical factor for APTSCs that exhibit inadequate photocurrent generation is that not all incident light can be fully utilized by the absorbers. Photons that do not enter the perovskite absorber layer due to interfacial reflection and parasitic absorption are categorized as external optical losses. The strategic mitigation of these losses aims to optimize the harvesting efficiency of photons at the absorber layer. Gao et al. calculated the implied photocurrent density of each layer via reasonable optical and electrical models for APTSCs, which is shown in Fig. 4a^28^. The optical losses mainly arise from reflection and parasitic absorption. The integrated reflection loss in APTSCs amounts to 8.81 mA cm^-2^ with distinct spectral dependence. In the ultraviolet range (300–400 nm), the primary source of loss is intrinsic reflection at the glass. In the visible light region (400-700 nm), cumulative reflections at multilayer interfaces dominate the loss mechanism, such as air/glass/ITO (refractive index *n* = 1.0 → 1.5 → 1.9) and perovskite/CTL (n = 2.1 → 1.5)^44^, collectively resulting in photon losses exceeding 5% within this spectral region. Extending into the NIR region (700-1200 nm), in addition to interfacial reflection losses, the NBG perovskite demonstrates reduced absorption (absorption coefficient ~10^4 ^cm^–1^ beyond 900 nm, Fig. 3d), which will be discussed in detail in Section 4.1. Consequently, 15–20% of incident photons undergo multiple reflections due to incomplete absorption. As for the parasitic absorption, it mainly comes from functional layers with suboptimal optical properties, including the front ITO (1.67 mA cm^–2^), Au (0.16 mA cm^–2^), PEDOT: PSS (0.57 mA cm^–2^), and Cu (0.28 mA cm^–2^). In addition to the unavoidable losses from the back-metal electrodes due to surface plasmon resonance, the remaining optical losses could be classified into reflection loss, and parasitic absorption from the front electrodes, CTL, and ICL^45–47^. This section will explore each of these losses in detail.

### Reflection of APTSCs

Reflection occurs at any interface with a difference in the refractive index of the two media, and the interfaces with significant refractive index differences lead to significant reflection losses. In APTSCs, reflection losses mainly occur at the back-metal electrode, the front glass/air interface, and the interfaces within the ICL^48^, as shown in Fig. 1e. The presence of the back-metal electrode will result in strong reflection at NIR region below the bandgap energy (>1.1 μm), which cannot be utilized by APTSCs. This reflection can only be reduced by further narrowing the bandgap of back sub-cell. However, achieving this in APTSCs poses significant challenges. The reflection occurring at the other two interfaces is the key to light management, as it can reduce the optical energy available to the two absorbers, ultimately leading to decreased photocurrent. To effectively mitigate the reflection loss of APTSCs, the design of anti-reflective layer becomes essential.

One type of anti-reflective layer uses a light-trapping structure to capture incident light from various angles, enhance scattering, and reduce light escape. This maximizes light absorption within the solar cell. Tavakoli and colleagues reported an anti-reflective layer composed of polydimethylsiloxane (PDMS) in front of glass. The PDMS layer was in the shape of a nanocone array (Fig. 4b), which generated light scattering. The nanocone array with an aspect ratio of 1.0 achieved 8% of reflectance reduction and improved the device performance from 12.06 to 13.14%^49^. This verified the feasibility of PDMS as an anti-reflective coating. Subsequently, various morphologies of PDMS were reported as the anti-reflective layers, such as inverted micro-pyramidal structure^50^, the epidermal texture of rose petals structure^51^, inverted hemispherical structure^52^, PDMS two-layer structure^53^, etc., proving their benefit to improve the device performance. In addition to organic polymer materials, inorganic materials can also play a role in reducing reflection. Wang et al. reported a mesoporous SiO_2_ anti-reflective coatings (Fig. 4c). The optical reflection loss of FTO/glass at 350–800 nm wavelength range could be reduced by adjusting the porosity and thickness of the SiO_2_ layer. The maximum achievable transmittance enhancement reaches 4 percentage points^54^.

Another strategy is to design an anti-reflective layer with suitable refractive index as a buffer layer, allowing light be refracted into the device several times to reduce reflection loss^55,56^. The LiF and MgF_2_ thin layers are widely used in photovoltaic devices because of their high transmittance and low refractive index. In 2018, Leijtens et al. reported a thermal evaporation method depositing a 190 nm-thick layer of MgF_2_ on the glass substrate to reduce reflection loss, resulting in enhanced absorption in the 680–920 nm wavelength range, which is shown in Fig. 4d^57^. Similarly, the LiF layer has also been reported to be used to the air/glass interface and the perovskite/C_60_ interface in ICL to improve device performance^58^.

Aforesaid two anti-reflective strategies for single-junction solar cells can also be applied to APTSCs. However, different from single-junction configurations, the introduction of these anti-reflective layers would result in photon redistribution within two sub-cells of APTSCs, disturb the previous photon balance, and complicate the optical modulation^59^. Different type, structure and thickness of anti-reflective layers will strongly impact their anti-reflective properties, which is specifically reflected in the amplitude and wavelength range of increased absorption. For 2 T tandem configurations, meticulous design of the anti-reflective layer is essential to ensure current-matching between the two sub-cells.

### Transparent conductive substrate

Parasitic absorption, occurring along the light incident path, refers to the absorption of photons by inactive layers (including electrodes, CTLs and ICLs) in solar cells, which does not contribute to the photocurrent. The parasitic absorption will lead to a decreased *J*_sc_ and PCE with reduced photons provided for the active layer. Electrodes, here primarily the front electrodes (such as ITO and fluorine-doped tin oxide, FTO), are the first layer face to light incidence in solar cells, attracting particular attention to the optical transmission enhancement. Many design strategies of transparent conductive substrate were approached from the perspective of structure and material to further reduce parasitic absorption.

Currently, transparent conductive oxides (TCO) used in PSCs primarily consist of ITO and FTO. Specifically, ITO demonstrates excellent optoelectronic characteristics with low sheet resistance (10–50 Ω/sq) and high transmittance (>85%), making it a more preferred choice for high-efficiency laboratory-scale devices. However, its industrial application is constrained by the scarcity of indium reserves and consequent high material costs. In contrast, FTO has become the mainstream commercial option due to its superior thermal stability and cost-effectiveness (sheet resistance 8–15 Ω/sq, transmittance 80–85%). Its optical performance degrades with increased film thickness and its fabrication requires high-temperature processing. Some light management strategies regarding the modification of ITO and FTO are being explored. Yu et al. reported an electrochemical etching method for treating commercial FTO glass^60^. This morphological optimization enhances light confinement and facilitates charge extraction, thereby significantly reducing the optical losses (Fig. 4e). Seok et al. developed a composition-engineered ITO (CE-ITO)^61^. Compared with commercial ITO (10 at. % Sn-doped), the CE-ITO showed lower Sn-doping (7.5 at. % Sn-doped) and presented a large columnar structure with higher light transmittance in the active layer (shown in Fig. 4f), thus enabling perovskite devices to achieve better efficiency and stability.

The selection of transparent front electrode constitutes an effective strategy as well. Schultes and co-workers described a pathway for the preparation and integration of H-doped indium oxide electrodes (IO:H) in perovskite solar cells. The IO:H was highly NIR transparent and conductive, reducing the integrated NIR absorption in the top sub-cell from more than 20% to less than 7% and avoiding current density losses of over 2 mA cm^–2^, which is shown in Fig. 4g^62^. The IO:H electrodes were also utilized in monolithic all-perovskite tandem solar cells and modules to improve the light utilization of Pb-Sn NBG sub-cells^63^. Additionally, the Zr-doped indium oxide (IZrO) showed a similar effect and was used in 4 T perovskite-based tandem solar cell^64,65^.

Alternative TCO materials, such as aluminum-doped zinc oxide (AZO, ~10^–3^ Ω cm)^66–69^ and gallium-doped zinc oxide (GZO, ~10^–4^ Ω cm)^70,71^, show promise but face inherent limitations. Notably, AZO suffers from interfacial instability under humid conditions due to hydrolysis susceptibility^72^, whereas GZO’s commercialization potential is constrained by the volatile pricing of gallium raw materials. These limitations underscore three interrelated challenges in TCO development. Firstly, achieving high conductivity without compromising optical transparency remains a fundamental materials science dilemma. Secondly, the strategic about critical elements like indium and gallium necessitates the exploration of earth-abundant alternatives. And finally, the mechanical robustness required for flexible devices demands addressing both the intrinsic brittleness of ITO and the dual sensitivity of AZO to thermal/humidity stresses. According to the three challenges, future research may focus on optimizing PV performance: (I) multi-element doping strategies (e.g., Ga/Al co-doped ZnO), (II) metal composite structures for enhancing light trapping and balancing photoelectrical performance, (III) low-temperature deposition processes compatible with flexible substrates, and (IV) exploration of indium-free and low-cost systems to improve sustainability and scalability.

### Transparent charge transport layer and interconnecting layer

Another parasitic absorption driven from the CTL and ICL should also be taken sufficiently into consideration. The optical design of the CTL requires low parasitic absorption to guarantee adequate the light capture of absorbers, where materials with serious parasitic absorption need to be avoided. The light management for ICL of APTSCs, in contrast to typical single-junction solar cells, faces multiple challenges beyond optical properties, including compatibility of processing technology and ability of carrier recombination. All of these factors impact the integration of ICL in monolithic tandem solar cells.

#### Transparent charge transport layer

There are four constituent parts of CTLs in 2J-APTSCs, namely ETL and HTL of WBG perovskite, ETL and HTL of NBG perovskite. The selection of materials for CTLs is extremely important because the three CTLs in the incident path need to be transparent. For example, Spiro-OMeTAD is a typical HTL for n-i-p structure devices, but it is unsuitable for p-i-n APTSCs cause it will absorb a large amount of photons in the ultraviolet region of the solar spectrum, resulting in severe optical loss^73^. Current APTSCs are more inclined to evade the n-i-p structure. In p-i-n structure, thermal evaporated C_60_ exhibits minor optical loss of around 0.05 mA cm^–2^. It is only 8.8% of the corresponding HTL loss (PEDOT: PSS, around 0.57 mA cm^–2^), especially at NIR region. Thus, it acts as appropriate ETLs for both WBG and NBG sub-cells (Fig. 4a).

The optimization of the HTLs for two sub-cells is more compatible and urgent. For WBG sub-cell, the HTL is usually adopted as PTAA or NiO, which is better for ultraviolet transmission. In addition, the discovery and application of SAMs in recent years have greatly promoted the development of APTSCs, such as [2-(9H-carbazol-9-yl)ethyl] phosphonic acid (2PACz), [4-(3,6-dimethyl-9H-carbazol-9-yl)butyl]phosphonic acid (Me-4PACz), [2-(3,6-dimethoxy-9H-carbazol-9-yl)ethyl]phosphonic acid (MeO-2PACz), etc. By virtue of the monolayer assembly, this ultra-thin HTL (usually several nanometers to tens of nanometers) minimizes the optical loss of the PSCs, showing minimal parasitic absorption across the full wavelength of the solar spectrum while ensuring efficient electrical transmission^74^. SAMs prompt the development of the CTL of APTSCs, as shown in Table 2.

**Table 2:** The application of different SAMs in perovskite solar cells

Year	SAMs	Configuration	Performance				Ref.
			*V*_oc_ (V)	*J*_sc_ (mA cm^-2^)	FF (%)	PCE (%)	
2022	2PACz MeO-2PACz	Flexible: PET/ITO/2PACz+MeO-2PACz/FA_0.8_Cs_0.2_PbI_1.95_Br_1.05_/C_60_/ALD-SnO_2_/Au/ PEDOT: PSS/FA_0.7_MA_0.3_Pb_0.5_Sn_0.5_I_3_/C_60_/BCP/Cu	2.00 2.02	15.8 15.7	78.3 74.1	24.7 (0.049 cm^2^) 23.5 (1.05 cm^2^)	Ref. ^154^
2022	2PACz	Flexible: PEN/ITO/2PACz/Cs_0.12_FA_0.8_MA_0.08_PbI_1.8_Br_1.2_/PCBM/ALD-SnO_2_/ITO/ PEDOT: PSS/(FASnI_3_)_0.6_(MAPbI_3_)_0.4_/C_60_/BCP/Cu	2.10	15.1	75.1	23.8	Ref. ^155^
2023	2PACz MeO-2PACz	Glass/ITO/NiO/2PACz+MeO-2PACz/CsPbI_3-x_Br_x_/C_60_/ALD-SnO_2_/Au/PEDOT: PSS/ FA_0.7_MA_0.3_Pb_0.5_Sn_0.5_I_3_/C_60_/BCP/Cu	2.00	16.1	79.6	25.6	Ref. ^156^
2023	4PADCB	Glass/ITO/4PADCB/FA_0.8_Cs_0.2_PbI_1.8_Br_1.2_/C_60_/SnO_2_/IZO/PEDOT: PSS/ FA_0.6_MA_0.3_Cs_0.1_Sn_0.5_Pb_0.5_I_3_/C_60_/SnO_2_/Cu	2.11	15.4	83.1	27.0 (1.044 cm^2^)	Ref. ^82^
2023	4dp3PACz	Glass/ITO/4dp3PACz/FA_0.8_Cs_0.2_PbI_1.8_Br_1.2_/C_60_/ALD-SnO_2_/IZO/PEDOT: PSS/ Cs_0.025_FA_0.475_MA_0.5_Sn_0.5_Pb_0.5_I_2.925_Br_0.075_/C_60_/BCP/Ag	1.76	18.1	79.2	25.2	Ref. ^157^
2023	MeO-2PACz Me-4PACz	Glass/ITO/NiO/MeO-2PACz+Me-4PACz/FA_0.8_Cs_0.2_Pb(I_0.62_Br_0.38_)_3_/C_60_/ALD-SnO_2_/ ITO NCs/MeO-2PACz+Me-4PACz/FA_0.7_MA_0.3_Pb_0.5_Sn_0.5_I_3_/C_60_/BCP/Cu	2.11 2.13	16.7 16.0	79.5 78.0	28.1 (0.049 cm^2^) 26.6 (1.044 cm^2^)	Ref. ^33^
2023	2 F	Glass/ITO/2 F/FA_0.8_Cs_0.2_PbI_1.8_Br_1.2_/C_60_/SnO_2_/IZO/PEDOT:PSS/2 F/ FA_0.6_MA_0.3_Cs_0.1_Pb_0.5_Sn_0.5_I_3_/C_60_/SnO_2_/Cu	2.13	15.5	82.4	27.2	Ref. ^81^
2022	VNPB	Glass/ITO/P1/NiO/VNPB/Cs_0.35_FA_0.65_PbI_1.8_Br_1.2_/C_60_/ALD-SnO_2_/Au/ PEDOT: PSS/FA_0.7_MA_0.3_Pb_0.5_Sn_0.5_I_3_/C_60_/ALD-SnO_2_/P2/ALD-SnO_2_/Cu/P3	8.1	3.60	76.8	22.5 (20.25 cm^2^)	Ref. ^158^
2022	2PACz	MgF_2_/Glass/IO: H/P1/2PACz/FA_0.8_Cs_0.2_I_1.8_Br_1.2_/LiF/C_60_/SnO_x_/ITO or Au/ PEDOT: PSS/Cs_x_(FA_0.83_MA_0.17_)_1−x_Pb_0.5_Sn_0.5_I_3_/PCBM/C_60_/BCP/P2/Cu/P3	13.3	2.02	71.0	19.1 (12.25 cm^2^)	Ref. ^63^
2023	2PACz MeO-2PACz	Glass/ITO/P1/NiO/2PACz+MeO-2PACz/Cs_0.35_FA_0.65_PbI_1.8_Br_1.2_/HF/ALD-SnO_2_/Au/ PEDOT: PSS/FA_0.7_MA_0.3_Pb_0.5_Sn_0.5_I_3_/HF/ALD-SnO_2_/P2/ALD-SnO_2_/Cu/P3	17.0	1.79	76.5	23.3 (20.25 cm^2^)	Ref. ^159^
2024	Me-4PACz	Glass/ITO/P1/NiO/Me-4PACz/Cs_0.35_FA_0.65_PbI_1.8_Br_1.2_/C_60_/ALD-SnO_2_/Au/ PEDOT: PSS/FA_0.7_MA_0.3_Pb_0.5_Sn_0.5_I_3_/C_60_/ALD-SnO_2_/P2/ALD-SnO_2_/Cu/P3	17.2	1.85	78.2	24.9 (20.25 cm^2^)	Ref. ^97^

Arrange in the following order: 2 T all-perovskite tandem cells and all-perovskite tandem modules

*ITO* indium tin oxide, *IZO* indium zinc oxide, *PEN* polyethylene naphthalate, *PET* polyethylene terephthalate, *PCBM* [6,6]-phenyl-C_61_-butyric acid methyl ester, *BCP* bathocuproine, *ALD* atomic layer deposition, *PEDOT* PSS, poly 3,4-ethylenedioxythiophene: polystyrenesulfonate

**Fig. 5:**
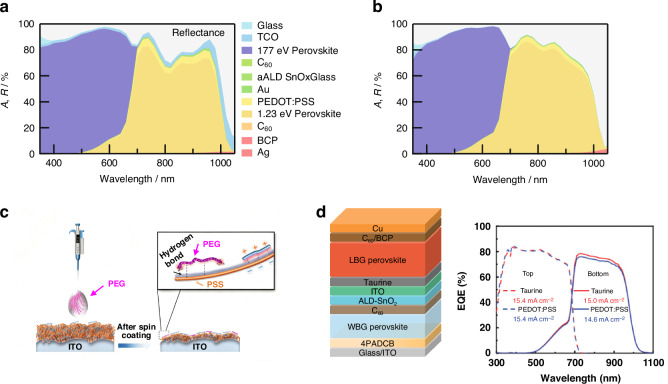
The optical loss caused by PEDOT: PSS. Simulated absorptance of APTSCs using **a** ITO as TCO and a 50 nm PEDOT: PSS HTL, and **b** IO: H as TCO and a 20 nm PEDOT: PSS HTL in the NBG sub-cell. **c** Schematic diagram of formation mechanism of PEG-PEDOT: PSS film. **d** Device structure and EQE curves of APTSCs with taurine. Panels **a** and **b** are reprinted from ref. ^45^ with permission from Wiley. Panel **c** is reprinted from ref. ^75^ with permission from Royal Society Chemistry. Panel **d** is reprinted from ref. ^77^ with permission from Wiley

For NBG sub-cell, PEDOT: PSS is the mostly used HTL but shows strongly parasitic absorption in NIR region, causing the photocurrent loss of the back sub-cell. Efforts have also been attempted to improve the photocurrent of the back NBG PSCs by thinning, removing, modifying or replacing PEDOT: PSS layer^45,75–77^. Datta et al. fabricated APTSCs on IO: H substrate using a thinned PEDOT: PSS layer (20 nm), and the current of APTSCs increased by 1.4 mA cm^–2^, as shown in Fig. 5a, b. Once optical losses resulting from the recombination layer were furtherly optimized, NBG sub-cell with a 600 nm-thick perovskite film could also achieve a high *J*_sc_ of 16.3 mA cm^-2^ in APTSCs, underscoring the importance of light management strategies^45^. The light management about ICL will be discussed in Section “Transparent interconnecting layer” specifically. Guo et al. reported a new strategy to treat PEDOT: PSS^75^. Aqueous polyethylene glycol (PEG) was introduced to thin the thickness of PEDOT: PSS, while residual PEG was able to bridge PEDOT: PSS with Pb-Sn perovskite (Fig. 5c). Pb-Sn perovskites showed both good optical and electrical properties with this treated HTL, displaying a PCE of 21.6% and a *J*_sc_ of 31.4 mA cm^–2^. Ma et al. proposed APTSCs with PEDOT: PSS-free configuration, as shown in Fig. 5d (left)^77^. 2-aminoethanesulfonic acid (i.e., taurine) was employed to improve optoelectronic properties of Pb-Sn perovskite with HTL-free contacts. The parasitic absorption in NIR region caused by PEDOT: PSS was removed (shown in Fig. 5d, right) and a PCE of 26.0% was achieved for APTSCs.

#### Transparent interconnecting layer

ICLs, the pivotal structure connecting the WBG sub-cell and NBG sub-cell in APTSCs, should not only block subsequent solvent flushing, but also achieve optical transparency especially for NIR light. ICLs are composed of CTLs on both sides, a solvent-barrier layer and a recombination layer. The parasitic absorption caused by CTLs has been discussed in Section “Transparent charge transport layer”, and the solvent-barrier layer (usually ALD-SnO_2_, deposited by atomic layer deposition) shows minor optical loss of 0.01 mA cm^–2^ (Fig. 4a). Therefore, we will focus on the structural evolution, optical properties and light management strategies for recombination layer in this section. Similar to the CTLs, strategies to reduce the optical losses of ICLs also include: thinning the thickness, selecting suitable materials with optical transmission, and removing materials with severe parasitic absorption.

**Fig. 6:**
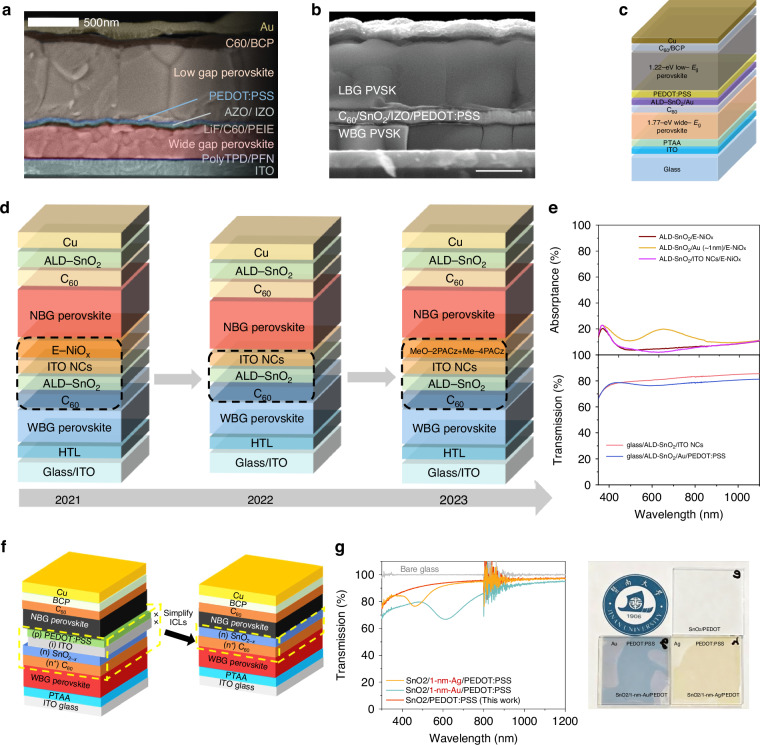
APTSCs with different ICL. **a** Thinning C_60_/PEIE/AZO/IZO/PEDOT: PSS as the ICL. **b** Simplified C_60_/SnO_2_/IZO/PEDOT: PSS as the ICL. **c** ICL with an ALD-SnO_2_ and ultra-thin Au recombination layer. **d** Structure schematic diagram of the APTSCs with ITO NCs-based ICLs. **e** Transmittance and absorbance spectra of the APTSCs with ITO NCs-based ICLs. **f** Simplified C_60_/SnO_1.76_ as the ICL. **g** Transmittance spectra of the ICLs with and without metal. Panel **a** is reprinted from ref. ^58^ with permission from Elsevier. Panel **b** is reprinted from ref. ^82^ with permission from Springer Nature. Panel **c** is reprinted from ref. ^83^ with permission from Springer Nature. Panel **e** is reprinted from ref. ^38^ and ref. ^43^ with permission from Wiley. Panel **f** is reprinted from ref. ^86^ with permission from Springer Nature. Panel **g** is reprinted from ref. ^87^ with permission from American Chemical Society

Initially, the ICL was introduced by Eperon et al. with a structure of [6,6]-phenyl-C_61_-butyric acid methyl ester (PCBM)/SnO_2_/zinc-tin-oxide (ZTO)/ITO/PEDOT: PSS, which is shown in Fig. 2e^27^. The buffer layer SnO_2_/ZTO/100-nm ITO layers protected the underlying perovskite completely from solvent damage, thus achieving a PCE of 16.9% for 0.2 cm^2^ devices and 13.3% for 1 cm^2^ devices. This ICL structure was gradually simplified into PCBM or C_60_/ALD-SnO_2_/ITO/PEDOT: PSS, and the combination of ALD-SnO_2_ and sputter-coated ITO was an effective way for APTSCs to realize high PCE^57,78,79^. Though thick ITO layer could block the solvent, it hindered the transmission of NIR light in the solar spectrum, showing a serious parasitic absorption for the back NBG perovskite. To avoid the use of thick metal oxide recombination layers, Palmstrom and colleagues developed a new ICL structure (Fig. 6a)^58^. They used an ultra-thin poly(ethylenimine) ethoxylated (PEIE) layer (1 nm) to induce the growth of a denser AZO layer, combined with an ultra-thin IZO (25 nm) tailor recombination layer, which reduced the lateral conductivity of the interlayer and optical loss. The APTSCs with the target ICL achieved a PCE of 23.1% on rigid substrates and 21.3% on flexible substrates. The ICL with IZO recombination layer exhibited better optical transmission than that with ITO, and was simplified into C_60_/SnO_2_/IZO/PEDOT: PSS in APTSCs as shown in Fig. 6b^80–82^.

Another alternative to recombination junctions is ultra-thin metal layers, such as Au and Ag. Lin et al. deposited an ultra-thin Au layer (1 nm) by thermal evaporation on the compact and robust ALD-SnO_2_ to facilitate electron-holes recombination, as shown in Fig. 6c^83^. It is important to note that the metal-based recombination junction is a double-edged sword. Although it is extremely thin and able to minimize recombination loss, it still causes significant parasitic absorption. The thickness of the metal layer is an important factor in its optical and electrical balance, which also brings about additional challenges for the current-matching of APTSCs. The ICL with superior optical properties remains the subject of ongoing research. A series of investigations about ICLs based on ITO NCs prepared by solution were conducted by Tan’s team, the ICL structures are shown in Fig. 6d^33,84,85^. The optical transmittance of ITO NCs-based ICL was better than that of Au/PEDOT: PSS-based ICL, achieving transmittance enhancement of around 4 percentage points, either as HTL or directly as recombination junction (Fig. 6e). Therefore, it was easier for ITO NCs-based ICL to achieve a higher photocurrent density. Further, by introducing two self-assembled carbazolyl molecules, MeO-2PACz and Me-4PACz, into ICL to tune the energy level of ITO NCs layer and extract holes quickly, a high PCE of 28.1% was achieved with a high *J*_sc_ of 16.7 mA cm^–2^ for 0.049 cm^2^ APTSCs^33^.

Researches on simplified metal-free ICLs are being impelling to refrain from serious parasitic absorptions caused by metal. Yu et al. acquired an incompletely oxidized SnO_1.76_ layer by adjusting the relative content of water in ALD^86^. The SnO_1.76_ layer exhibited an ambipolar carrier transport property due to the presence of Sn^2+^. The C_60_/SnO_1.76_ ICL formed Ohmic contacts with both WBG and NBG sub-cells, so the HTL and recombination layer could be omitted. The component of ICL thus could be simplified to just two layers (Fig. 6f), with reduced optical parasitic absorption. The APTSCs with C_60_/SnO_1.76_ ICL achieved a PCE of 24.4% and 22.2% with an aperture area of 0.059 cm^2^ and 1.15 cm^2^, respectively. Zhou et al. developed a metal-free ICL with an architecture of PCBM/ALD-SnO_2_/PEDOT: PSS^87^. They found that the Au layer (even only 1 nm) showed a significant optical loss at the full wavelength of 300–1200 nm, while the metal-free ICL showed a better transmittance (Fig. 6g), resulting in a transmittance of over 90% at NIR region and a PCE of 23.7% in APTSCs.

### Challenge of reducing optical losses for APTSCs

Overall, a fundamental understanding of the origins and location of optical losses in APTSCs is critical for developing effective light management strategies. Notably, light management about parasitic absorption in APTSCs is inherently more complex than in single-junction PSCs, due to the need to account for current matching and NIR light utilization. This complexity stems from the requirement to simultaneously optimize two distinct spectral regions: visible light absorption in the WBG sub-cell, and the harvesting of NIR photon in the NBG sub-cell. To achieve current matching, the solar spectrum must be carefully split so that photons are distributed in accordance with each sub-cell’s bandgap and current generation capability. The use of NBG perovskites extends the absorption range of APTSCs beyond the around 850 nm limit of single-junction PSCs, reaching up to 1050 nm. However, the harvesting of photons in the 700–1050 nm range remains inefficient. This is partly due to parasitic absorption in the NIR region caused by TCOs and charge transport layers. In addition, the intrinsic absorption of Pb-Sn mixed perovskites in this spectral range is limited. These issues will be further discussed in Section “Improving light utilization”.

To sum up, the roadmap for minimizing optical losses in APTSCs necessitates a dual-path optimization framework. Primarily, it may be inadequate for APTSCs to conduct conventional single-band (within visible region) anti-reflective strategies, but to develop spectrally engineered anti-reflective coatings with a covering NIR region (such as graded refractive index layers). Such a breakthrough requires in-depth understanding of material science including the fundamental physical properties, surface morphology and process deposition techniques. Concurrently, quantum efficiency enhancement in the NIR region necessitates systematic material innovation across device configuration. Specific strategies may include: (I) development of TCOs with reduced free carrier concentrations through co-doping optimization, (II) design of ultra-WBG CTLs exhibiting exceeding 90% NIR transparency via bandgap alignment engineering, or (III) implementation of functional ICLs incorporating spectrally selective reflectors. These advancements must be synergistically integrated to achieve <5% parasitic absorption in NIR spectral regions.

## Improving light utilization

Alongside reducing optical losses in APTSCs, improving light utilization is equally crucial. The former aims to increase incident photons, while the latter focuses on maximizing internal use of these photons through effective charge generation and collection. By employing effective strategies for light utilization, it is possible for APTSCs to minimize light escape and maximize photon usage^88^. In this section, we will outline several light management strategies to improve light utilization, including high quality NBG perovskite films for capturing low-energy photons (Section “More NIR light trapping: the role of mixed Pb-Sn perovskite”), micro-/nano-scale structure to enhance light path of photons (Section “More light confinement: micro-/nano-scale structure”), more junctions solar cell to minimize the losses of high-energy photons (Section “Less thermalization loss: multijunction solar cell”), and bifacial structures for capturing ambient light (Section “More light irradiation: ambient light capture and bifacial APTSCs”).

### More NIR light trapping: the role of mixed Pb-Sn perovskite

**Fig. 7:**
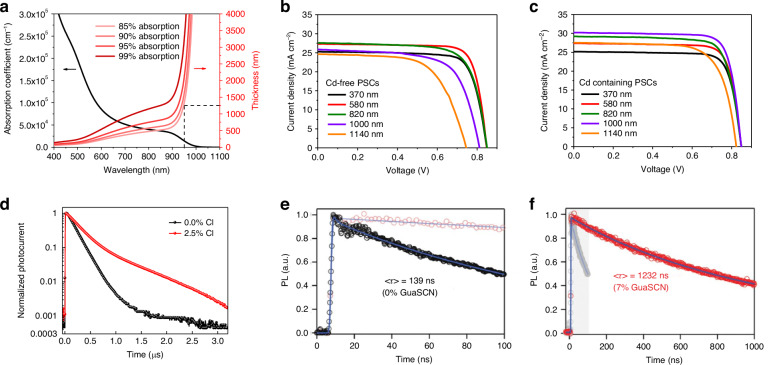
The thickness design for NBG PSCs. **a** Absorption coefficient of FA_0.7_MA_0.3_Pb_0.5_Sn_0.5_I_3_ and required thickness of the NBG perovskite films. **b** without Cd and **c** with 0.03 mol% Cd addictive in Pb-Sn perovskite films (FA_0.5_MA_0.45_Cs_0.05_Pb_0.5_Sn_0.5_I_3_) with different thickness. **d** Transient photocurrent decay (TPC) curves of NBG PSCs with 0.0% and 2.5% Cl. Time-resolved photoluminescence (TRPL) curves of NBG perovskites **e** without and **f** with 7% GuaSCN additive. Panels **b** and **c** are reprinted from ref. ^31^ with permission from Springer Nature. Panel **d** is reprinted from ref. ^94^ with permission from Springer Nature. Panels **e** and **f** are reprinted from ref. ^95^ with permission from AAAS

The integration of NBG perovskites into APTSCs enables the harvesting of NIR photon, extending the spectral utilization range beyond 900 nm and highlighting the competitive advantage of APTSCs. To optimize their light-trapping efficacy, we systematically investigated the absorption characteristics of FA_0.7_MA_0.3_Pb_0.5_Sn_0.5_I_3_-perovskites (Fig. 7a). NBG perovskites exhibited pronounced thickness-dependent absorption profiles, with absorption coefficients decreasing sharply in the NIR regime (particularly below 10^4 ^cm^–1^ at wavelengths exceeding 900 nm). This suboptimal absorption led to multi-pass photon reflections, manifesting as severe external quantum losses through radiative recombination channels. Our quantitative analysis reveals that increasing NBG perovskite layer by 200 nm enhances 950 nm photon capture efficiency by 5% (Fig. 7a), demonstrating the critical role of thickness in mitigating external quantum losses associated with low-energy photon absorption in APTSCs^30^. The similar absorption effects can be seen in other bandgap perovskites as well^89^.

However, the increase in absorber thickness brings about a significant challenge for efficient carrier transport. Effective charge collection necessitates the diffusion length (*L*_d_) of minority carrier within the perovskite layer at least exceed the absorber thickness^90,91^. For Pb-Sn NBG perovskite with a target thickness (*d*) of 1 μm, the corresponding diffusion length should also be greater than 1 μm. A summary of the high-PCE NBG devices (PCE > 20%, as detailed in Table 3) reveals that they typically exhibit *L*_d_ values over 2 *d*. Those devices achieving PCE > 22% have even demonstrated *L*_d_ values up to 5 *d*. Additionally, the carrier mobility of Pb-Sn perovskite generally ranges from 0 to 30 cm^2 ^V^–1^ S^–1^, which is lower than that of pure-Pb perovskite, ranging from several tens to hundreds^92,93^. This reduced mobility may be attributed to the higher defect density within the perovskite layer due to the oxidation Sn^2+^, thereby limiting the enhancement of *L*_d_ (since *L*_d_∝$\sqrt{\mu \tau }$). When *L*_d_ is insufficient (e.g., *L*_d_ < 2 *d*), increasing the absorber thickness leads to a decline in internal quantum efficiency (IQE) due to enhanced recombination. In essence, while thicker films can absorb more NIR light, they fail to effectively convert these captured photons into usable photogenerated carriers, resulting in suboptimal photovoltaic performance.

**Table 3:** Charge-carrier mobility, lifetime and diffusion length values for Pb-Sn perovskite

Year	Absorber thickness, *d* (μm)	Mobility, μ (cm^2^ V^–1^ S^–1^)	Diffusion lifetime, τ (ns)	Diffusion length, *L*_d_ (μm)	*L*_d_ / *d*	PCE (%)	Ref.
2019	1.0	1.98 (*e*) 3.86 (*h*)	1450	2.72 (*e*) 3.80 (*h*)	2.72 (*e*) 3.80 (*h*)	20.3	Ref. ^31^
2019	1.0	1.97*	1232	2.50	2.50	20.5	Ref. ^95^
2019	0.86	79 (*Σ*)	43.2	2.99 (*Σ*)	3.48	21.1	Ref. ^83^
2020	0.85	63 (*Σ*)	188	5.52 (*Σ*)	6.49	21.7 (0.049 cm^2^) 18.8 (1.05 cm^2^)	Ref. ^96^
2022	1.2	11.7	966	5.40	4.50	22.2	Ref. ^29^
2023	0.60	0.31 (*e*) 2.1 (*h*)	2430	1.39 (*e*)* 3.62 (*h*)*	2.31 (*e*) 6.03 (*h*)	22.6	Ref. ^160^
2023	0.88	10.12	2878	8.67	9.85	22.2	Ref. ^161^
2025	0.90	~ 20	550	5.32*	5.91	23.2	Ref. ^162^

a) The asterisks are the missing values in the reference and are calculated through ${L}_{d}=\sqrt{\mu {k}_{B}T\tau /e}$, where$\,\mu$ is the carrier mobility, ${k}_{B}$ is the Boltzmann’s constant, $T$ is the temperature in K,$\,\tau$ is the bulk carrier lifetime, and $e$ is the elementary charge;

b) *e* within the bracket is the electron, *h* is the hole, and *Σ* is the sum over both

Yang et al. investigated the absorption properties of FA_0.5_MA_0.45_Cs_0.05_Pb_0.5_Sn_0.5_I_3_ perovskite with a bandgap of 1.22 eV^31^. They found that thickness of NBG perovskite films should reach up to 1 μm to exhibit satisfactory absorption in the NIR region mentioned in Fig. 4d. However, because of the poor carrier diffusion length in the perovskite bulk (0.49 ± 0.10 μm), the NBG PSCs with 1 μm films showed decreased performance compared to that with 580 nm films (Fig. 7b). By adding 0.03 mol% Cd^2+^ into Pb-Sn perovskite precursors, the carrier diffusion length was increased to 2.72 ± 0.15 μm and the optimal perovskite thickness was increased to 1 μm. The PCE of champion device with 1000-nm-thick absorber film reached 20.3% (Fig. 7c). Anion additives can also perform well in enhancing the crystal quality of Pb-Sn perovskite. Zhao et al. reported a bulk-passivation strategy via incorporation of Cl^-^ to enlarge grains and increase carrier diffusion length, as shown in Fig. 7d^94^. The target NBG PSCs and APTSCs achieved a champion PCE of 18.4% and 21.0%, respectively. Tong et al. used the guanidinium thiocyanate (GuaSCN) additive to improve the structure and photoelectric properties of Pb-Sn perovskite films^95^. The films exhibited a significantly improved carrier lifetime of over 1 μs and a long carrier diffusion length of 2.5 μm (Fig. 7e, f). These improved properties enable the NBG PSCs and APTSCs to achieve a PCE of 20.5% and 23.1%, respectively.

**Fig. 8:**
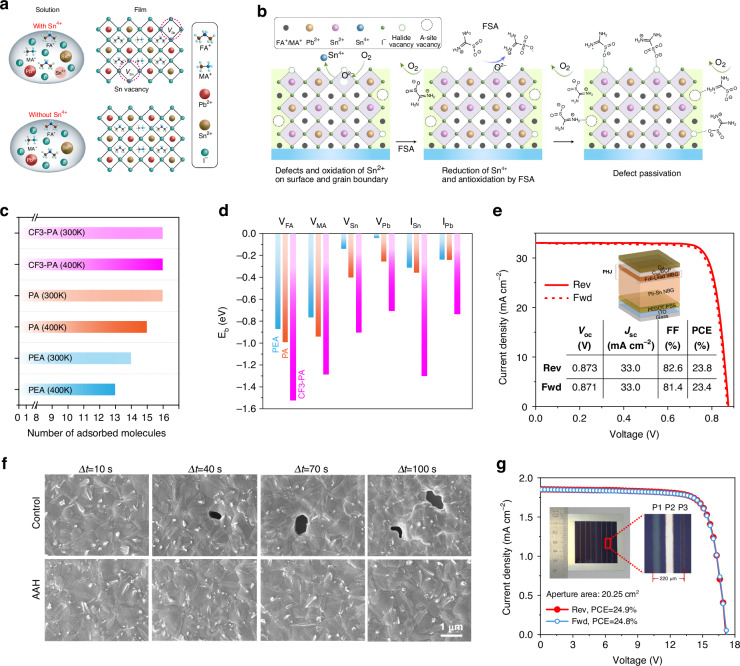
APTSCs with improved NBG perovskite films. **a** Illustration of the formation of Sn vacancies in mixed Pb-Sn perovskite due to the presence of Sn^4+^ in the precursor solution and the suppression of Sn vacancy formation in TRP perovskite because of the absence of Sn^4+^. **b** Schematic illustration of antioxidation and defect passivation at grain surfaces of mixed Pb-Sn perovskite films enabled by FSA. **c** The number of absorbed molecules for CF3-PA, PA and PEA at temperatures of 300 and 400 K. **d** The binding energy (E_b_) between passivators and various acceptor-like defects. **e** *J*-*V* curves and schematic illustration of APTSCs with PHJ. **f** Crystal growth process of Pb-Sn perovskite film without and with AAH additive. **g** *J*-*V* curves of all-perovskite tandem modules with AAH additive. Panel **a** is reprinted from ref. ^83^ with permission from Springer Nature. Panel **b** is reprinted from ref. ^96^ with permission from Springer Nature. Panels **c** and **d** are reprinted from ref. ^29^ with permission from Springer Nature. Panel **e** is reprinted from ref. ^35^ with permission from Springer Nature. Panels **f** and **g** are reprinted from ref. ^97^ with permission from AAAS

Our team was also committed to developing high-quality Pb-Sn perovskite films. To inhibit Sn^2+^ oxidation in perovskite precursors, metallic Sn powder was introduced into perovskite precursors^83^. The perovskite films with Sn-reduced precursor solution strategy increased the carrier diffusion length from 0.75 to 2.99 μm due to fewer defects (mainly Sn vacancies, shown in Fig. 8a). As a result, the Sn-Pb PSCs using Sn powder in the precursor solution achieved over 21% PCE with a Pb-Sn perovskite thickness of 860 nm. Further, the zwitterionic antioxidant formamidine sulfinic acid (FSA) was used to inhibit the oxidation of Sn^2+^ and to passivate the defects on the grain surface of the mixed Pb-Sn perovskite film (Fig. 8b), resulting in a single-junction PSC with a PCE of 21.7%^96^.

Although we need to focus on the crystal quality of Pb-Sn perovskite, defects on the top surface should also not be ignored. In 2022, Lin et al. reported a strong absorption molecule 4-trifluoromethyl-phenylammonium (CF3-PA) to deeply passivate the film defects^29^. Compared to other aromatic ammonium ions, CF3-PA presented a stronger tendency to anchor on the perovskite surface during film formation, while increasing the binding energy to the negatively charged defects (Fig. 8c, d). The Pb-Sn perovskite film with CF3-PA thus showed less defects and longer carrier diffusion length than that without additives or with other aromatic ammonium ions. The Pb-Sn PSCs with CF3-PA allowed 1.2 μm thick films to achieve a high PCE of over 22%. To further reduce surface defects, Lin et al. developed an immiscible-phase 3D/3D bilayer perovskite heterojunction (PHJ) with a type II band structure at the Pb-Sn perovskite/C_60_ interface, which ensured that the charge transporting losses were minimized^35^. The NBG PSC with a 1.2-μm-thick absorber achieved a high *V*_oc_ of 0.873 V and a high FF of 82.6% (Fig. 8e), showing that the Pb-Sn perovskite exhibited a high crystal quality.

As for defects in the bottom interface of Pb-Sn perovskite, Gao et al. investigated the crystallization of Pb-Sn perovskite during scalable fabrication^97^. They found that the nonuniform crystallization and inferior buried perovskite interface seriously hindered the breakthroughs of the all-perovskite tandem modules. Thus, a dopant, aminoacetamide hydrochloride (AAH), was used to homogenize crystallization, extend the processing window for blade-coating Pb-Sn perovskite films, and passivate the buried perovskite interface (Fig. 8f). The all-perovskite tandem module with this uniform, high-quality and 1-μm-thick Pb-Sn perovskite films achieved a high certified PCE of 24.5%, with an aperture area of 20.25 cm^2^ (Fig. 8g).

### More light confinement: micro-/nano-scale structure

Micro-/nano-scale structure is a feasible solution for APTSCs due to increasing path length of photons. There have been numerous successful cases in monolithic perovskite/c-Si tandem configurations. One of the most popular strategies is to use textured c-Si to reduce light scattering loss^98,99^. Hou et al. systematically investigated the light-trapping superiority of textured-silicon^100^. Their quantification revealed a progressive weighted reflectance reduction from 36% to 21% with texture dimensions from planar to 200 nm features, reaching an ultralow 15% weighted reflectance at 2 μm patterning. This geometric optimization effectively elongates photon dwell pathway. It shows that textured structure also affects light absorption, either absorber or functional layer. However, typical APTSCs now exhibit a planar structure, and it is not practical to fabricate perovskite into a textured pyramid structure similar to c-Si. An alternative scheme is to fabricate micro-/nano-scale structure to complicate light pathway and light scattering, achieving more light trapping sites, including glass/electrode substrates, functional layers or perovskite itself. However, given the complex structure and restrictions in the preparation technology of APTSCs, this aspect of studies is very challenging, so the reported studies on APTSCs are very limited.

**Fig. 9:**
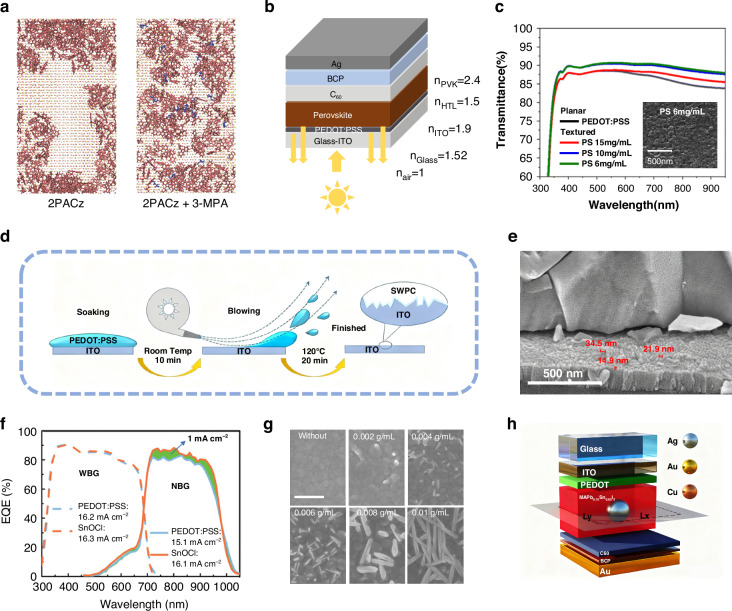
The design for micro-/nano-scale structure of APTSCs. **a** Top view of equilibrated molecular representations of control and mixed systems. 2PACz and 3-MPA are shown in pink and blue, respectively; Sn and O atoms, shown in the background, are depicted in yellow and red, respectively. **b** Illustration of the p-i-n device structure and the reflective indices. **c** Transmittance of glass with planar and textured PEDOT: PSS HTL processed with different concentrations of PS spheres. **d** Schematic diagram of SWPC-PEDOT: PSS deposition method. **e** Top-view SEM image of SnOCl HTL with textured morphology. **f** EQE response of APTSCs with PEDOT: PSS-based and SnOCl-based ICLs. **g** Top-view SEM images of CPRA at different HCOOCs concentrations with the Scale bar of 1 µm. **h** Scheme of the single-junction plasmonic solar cell system employed in the simulations. Panel **a** is reprinted from ref. ^101^ with permission from Springer Nature. Panels **b** and **c** are reprinted from ref. ^44^ with permission from Royal Society Chemistry. Panel **d** is reprinted from ref. ^102^ with permission from Elsevier. Panels **e** and **f** are reprinted from ref. ^103^ with permission from Wiley. Panel **g** is reprinted from ref. ^105^ with permission from Elsevier. Panel **h** is reprinted from ref. ^106^ with permission from American Chemical Society

From the perspective of light management, APTSCs deposited on flat and smooth ITO substrates experience more serious light loss than those on textured FTO substrates. Due to the presence of the vertebral body, the textured FTO substrate is able to extend the light path of the incident light and minimize the reflection loss. Park et al. studied the molecular dynamics of phosphoric acid deposition on textured substrate^101^. Their simulations showed that 2PACz deposited on textured SnO_2_ formed polymers and significant phase segregation due to agglomeration. However, the introduction of 3-mercaptopropionic acid can decompose these 2PACz clusters, especially on the textured substrates, and improve the surface coverage of 2PACz (as shown in Fig. 9a). This study revealed possible problems with the deposition of phosphate molecules on textured substrates, which presented implications for APTSCs.

The textured functional layers bring opportunities for photon absorption of absorbers. Hsu et al. investigated the optical loss of PEDOT: PSS/Pb-Sn perovskite interface^44^. They believed that the low refractive index of planar PEDOT: PSS and the high refractive index of Pb-Sn perovskite were the main reasons for the internal reflection loss (Fig. 9b). Therefore, they created a textured surface on PEDOT: PSS through the application of a polystyrene (PS) sphere patterning methods. The devices with textured PEDOT: PSS showed a 7% increase in photocurrent compared to that with planar PEDOT: PSS (Fig. 9c). Xu et al. reported a method for preparing a pinhole-free monolayer PEDOT: PSS mesh film (SWPC-PEDOT: PSS), with the process shown in Fig. 9d^102^. Compared to the pristine PEDOT: PSS, SWPC-PEDOT: PSS presented a negligible optical interference and kept the high coverage, thus contributing to improving PCE. Yu et al. replaced PEDOT: PSS by SnOCl with a textured morphology as the HTL of Pb-Sn perovskite (Fig. 9e)^103^. The mixed neutral PEDOT/SnOCl ICL for APTSCs exhibited lower optical loss in the NIR region of the spectrum, as shown in Fig. 9f.

The morphology of the perovskite itself also has an impact on the light absorption. The perovskite film with a 2D inverse opal structure, as reported by Meng et al. introduced a novel concept for the morphology of perovskite^104^. Gao et al. synthesized a Cs-based perovskite recrystallization array (CPRA) with excellent crystallinity on the surface of WBG perovskite films to enhance the light capturing ability of planar PSCs, as shown in Fig. 9g^105^. Due to the wide wavelength absorption range and special anti-reflective structure of the CPRA, the PSCs showed a highly optimized spectral response, and the PCE increased from 19.92% to 23.23%. Bueno et al. investigated near- and far-field plasma effects to locally enhance the light absorption of NIR photons^106^. Their simulations showed that adding 120 nm silver particles into the Pb-Sn perovskite layer with a volume concentration of 3.1% could increase the absolute PCE of the APTSCs by 2%, as shown in Fig. 9h. In order to achieve a similar enhancement in the control APTSCs, it would be necessary to double the thickness of the Pb-Sn perovskite layer. This study presented a promising avenue for APTSCs to realize ultrathin absorbers with optimized charge carrier collection.

Nevertheless, the preceding functional layer (or absorber) is textured, the subsequent nonconformal processing may not preserve this texture. In APTSCs, maintaining or re-establishing a textured morphology to enhance the harvesting of photons in the back sub-cell remains a significant challenge. In addition, the coupled effects of texture dimensional scaling and periodic arrangement on photon/charge carrier dynamics remain systematically unexplored. Implementing machine learning-assisted topology optimization with optical and drift-diffusion modeling offers an approach to establish structure-property relationships, potentially revealing optimal designs that maximize both light trapping and charge extraction.

### Less thermalization loss: multijunction solar cell

Compared with 2J-APTSCs, triple-junction APTSCs (3J-APTSCs) are able to further reduce thermalization heat by connecting a sub-cell with higher bandgaps (>1.9 eV). Through precise bandgap engineering of the absorption layer and thickness modulation of functional layers, the optimized 3J-APTSCs demonstrated superior spectral response within high-energy photon range, attaining 90% quantum efficiency at 400 nm and approaching 100% at 500 nm, which represented a 28.6% enhancement compared to 2J-APTSCs (70% at 400 nm) under the same identical optimized model^107^. Therefore, 3J-APTSCs have great potential in making full use of absorbed photons, achieving high PCE and improving economic benefits.

**Fig. 10:**
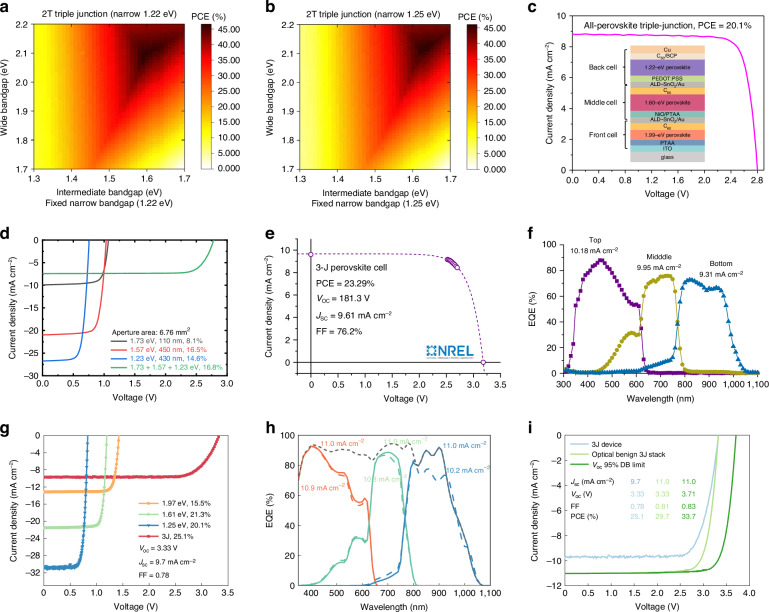
3J-APTSCs. Bandgap calculation for 3J-APTSCs by connecting the bandgap of **a** 1.22 eV and **b** 1.25 eV NBG sub-cells. **c** *J*-*V* curve of 3J-APTSCs constructed by Xiao et al. **d** *J*-*V* curve of 3J-APTSCs constructed by Wang et al. **e** *J*-*V* and **f** EQE curves of 3J-APTSCs constructed by Wang et al. **g** *J*-*V* curve of 3J-APTSCs constructed by Wang et al. **h** EQE spectra of each sub-cell and total EQE spectrum, and **i** *J*-*V* curves of 3J-APTSCs with optical design. Panels **a** and **b** are reprinted from ref. ^109^ with permission from Royal Society Chemistry. Panel **c** is reprinted from ref. ^111^ with permission from American Chemical Society. Panel **d** is reprinted from ref. ^112^ with permission from Springer Nature. Panels **e** and **f** are reprinted from ref. ^113^ with permission from Springer Nature. Panels **g-i** are reprinted from ref. ^114^ with permission from Springer Nature

According to the mentioned design principles and light management strategies in Section “Optical design principle for APTSCs”, the bandgap and thickness of sub-cells in 3J-APTSCs need to be redesigned to reduce optical loss. Through modeling, Hussain et al. analyzed that when the bandgap of the three sub-cells achieved 1.94, 1.55 and 1.22 eV respectively, the PCE could reach 28.38%^108^. Lim and co-workers gave the calculation results of the theoretical PCE limit, as shown in Fig. 10a, b^109^. When a bottom bandgap of 1.22 eV was used, an intermediate bandgap of ~1.60 eV and a wide bandgap of ~2.10 eV combination can achieve over 46% PCE (Fig. 10a). And when a bottom bandgap of 1.25 eV was used, an intermediate bandgap of ~1.65 eV and a wide bandgap of ~2.13 eV combination can achieve over 45% PCE (Fig. 10b). However, although there are a large number of studies on the bandgap of 3J-APTSCs, there are still few researches on the optimal thickness and optical design, which result in serious current loss in 3J-APTSCs.

In fact, the configuration of 3J-APTSCs is grounded on that of 2J-APTSCs. In 2019, McMeekin et al. reported the first 3J-APTSCs^110^. The devices used the sub-cells with n-i-p structure and the functional layers demonstrated serious parasitic absorption, thus showing a suboptimal photovoltaic performance. Their model forecasted that by optimizing the optical and electrical parameters of the structure, 3J-APTSCs could achieve a *V*_oc_ of 3.16 V and a PCE of 26.7%. In 2020, Xiao et al. achieved a 3J-APTSCs PCE of over 20% by optimizing the p-i-n bandgaps of the sub-cells (1.99/1.60/1.22 eV) to make full use of the solar spectrum and connecting them with the ALD-SnO_2_/Au ICLs to reduce electrical losses, as shown in Fig. 10c^111^. In the same year, Wang et al. used 3J-APTSCs with similar structure but sub-cells bandgaps of 1.73/1.57/1.23 eV, achieving a PCE of 16.8%, as shown in Fig. 10d^112^. In 2023, Wang et al. used a Rb/Cs mixed-cation inorganic perovskite with a bandgap of approximately 2.0 eV as the top WBG cell, and achieved a 3J-APTSCs PCE of 24.3% (certified PCE of 23.3%, shown in Fig. 10e)^113^. The integrated *J*_sc_ values for the 2.0-eV, 1.6-eV and 1.22-eV sub-cells from EQE measurements were 10.18, 9.95 and 9.31 mA cm^−2^, respectively, exhibiting an obvious current-mismatch (Fig. 10f). Wang et al. found that a diammonium halide salt, propane1,3-diammonium iodide, introduced into the WBG film, could improve halide homogenization, leading to a record *V*_oc_ of 1.44 V^114^. By connecting 1.97/1.61/1.25 eV perovskite, the 3J-APTSCs achieved a PCE of 25.1% (Fig. 10g), but a large current mismatching of 3.6 mA cm^–2^ occurred because of the limited current of the middle sub-cell. Their reconstructed optical model showed a potential *J*_sc_ values of 11.0 mA cm^-2^ and a potential PCE of 29.7% (Fig. 10h, i) by fine tuning the thickness of perovskite absorbers and implementing more optically transparent layers, which indicating the importance of optical design for efficient 3J-APTSCs.

Although 3J-APTSCs have made great breakthroughsrecently, the device performance still lags behind that of 2J-APTSCs, which may be related to the following problems. (I) Current matching. The highest PCE of 3J-APTSCs at present still showed serious current-matching loss^114^, implying an uneven distribution of photons in the sub-cells due to the unclear mechanism of thickness-matching among the three sub-cells. Thus, it is urgent for 3J-APTSCs to establish a more specific optical model and make more accurate theoretical predictions based on electrical parameters. (II) ICLs. In more intricate configurations, two ICLs of 3J-APTSCs suffer from more serious optical parasitic absorption, such as double utilization of the recombination Au layer. This phenomenon suggests that ICLs designs tailored for 2J-APTSCs might not function well in the 3J-APTSCs, and necessitate meticulous attention especially the one between the top and middle sub-cells. (III) Br-rich perovskite. Compared with the middle and the back sub-cells, researches on the top sub-cells (absorber layer of 2.0 eV perovskites) are still too insufficient to achieve perfect current-matching, such as serious interfacial non-radiative recombination and long-term phase stability under prolonged illumination, limiting their photovoltaic performance.

### More light irradiation: ambient light capture and bifacial APTSCs

**Fig. 11:**
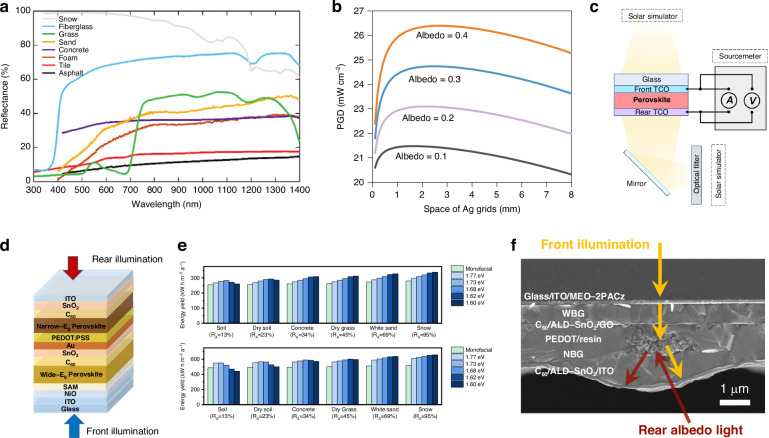
Bifacial APTSCs. **a** Reflectance spectra of common ground materials. **b** PGDs (power-generation density) of a bifacial module under different albedos based on a monofacial one. **c** Schematic diagram of measurement installation for bifacial illumination using two simulators. **d** Schematic illustration of bifacial monolithic APTSCs. **e** Energy yield calculation of monofacial and bifacial tandems with various environment conditions. **f** SEM image of a bifacial APTSCs with a light-trapping resin particle layer. Panel **a** is reprinted from ref. ^116^ with permission from Wiley. Panel **b** is reprinted from ref. ^117^ with permission from Springer Nature. Panel **c** is reprinted from ref. ^115^ with permission from Elsevier. Panels **d** and **e** are reprinted from ref. ^123^ with permission from Springer Nature. Panel **f** is reprinted from ref. ^124^ with permission from AAAS

In practical applications, the fraction of reflected sunlight by the environment provides the rear-side irradiance of bifacial solar cells^115^. Figure 11a shows the reflection spectra of common ground materials under normal incidence, according to the National Aeronautics and Space Administration’s (NASA’s) ECOSTRESS Spectral Library^116^. Urban materials show a broad range of reflectivity. Conventional concrete typically exhibits 30-35% solar reflectance, while glass fiber composites demonstrate exceptionally high reflectivity (68-72% across 300-1400 nm spectrum). Although vegetative surfaces display spectral selectivity, they still achieve 48-52% reflectance in 300-1100 nm range. Comparatively, desert regions maintain a broadband albedo of 40-45% due to quartz-rich sand composition (85-92% SiO_2_ content), though diurnal variations exceed 12% from solar zenith angle effects. Albedo is generally defined as a ratio of the reflected radiation flux and the incident radiation flux. When the albedo is high, the number of photons from the rear side illumination becomes significant to the total power output of a bifacial solar cell (Fig. 11b)^117^. In practical scenarios, it is essential for PSCs to focus on the incident light originating from the back and maximize its utilization.

Employing bifacial designs with transparent conductive back electrodes, harvesting reflected light and diffuse light from the rear side, is an excellent choice to make full use of the ambient light^118–122^. The power gain from a bifacial single-junction solar cell compared with a monofacial equivalent depends on the amount of light incident on the rear surface. For bifacial 2 T tandem solar cells, the power gain also relies on the current matching of sub-cells under rear light incident^122^. Figure 11c depicted the bifacial architecture for PSCs, holding potential to offer further increases in the energy yield of perovskite photovoltaics^115^. To evaluate the output power density in lab, the bifacial PSC was placed between two AM 1.5 G solar simulators. The front illumination was set to a standard intensity of 100 mW cm^–2^, and the rear illumination was adjustable between 0 and 50 mW cm^–2^ to simulate various albedo light^115^. With the implementation of a bifacial structure, the light utilization of the device is enhanced, resulting in a total power output that is the cumulative sum of conversion power generated from the both sides of the incident light.

Recent advances in device process and optical optimization enable the 2 T APTSCs to achieve really high PCE of 30.1%^10^. Fabricating bifacial APTSCs by improving light utilization enable higher output power density than their monofacial counterparts. Li et al. demonstrated the bifacial monolithic APTSCs for the first time by replacing the rear metal electrodes with transparent conduction oxide electrodes (Fig. 11d). The back NBG sub-cell absorbed virtually all the albedo illumination, leading to severe current mismatching. Bandgap engineering of front WBG perovskite sub-cell with different absorption spectrum range should be elaborately deployed to adapt various rear illumination conditions. The champion bifacial APTSCs achieved a high output power density of 28.51 mW cm^−2^ under 30 mW cm^−2^ rear illumination. Further energy yield calculation proofed the substantial energy yield gain for bifacial tandems under six kinds of ground albedo for two climatic conditions (Fig. 11e)^123^. The transparent electrode for bifacial tandems runs risks of reducing photon path length of NBG sub-cells without the reflection on the rear metal electrode. Embedding a light-scattering micrometer-sized particle layer into perovskite as light trapper effectively increase absorptance by 5% to 15% in the NIR region. Moreover, using a nonacidic PEDOT: PSS HTL stabilized the hole-extraction interface by avoiding proton-accelerated formation of iodine. Combining the two strategies together increased efficiency of semitransparent Pb-Sn cells from 15.6 to 19.4%, enabling fabrication of efficient bifacial APTSCs with an equivalent efficiency of 29.3% under 30% of albedo light (Fig. 11f)^124^. Bifacial APTSCs with “substrate configuration” have also been reported, holding potential to be fabricated on a large variety of inexpensive substrates, such as transparent plastic. A solution-processed NBG PSC was deposited on TCO-coated glass, following which all of the other layers were processed using vacuum sublimation, deserved to be mentioned that not only the CTLs and ICLs, but also the WBG perovskite films. This kind of bifacial APTSCs obtained promising efficiency up to 20%, surpassing the efficiency of the corresponding single-junction devices with substrate configuration^125^. It is noteworthy that although the TCO transparent rear electrodes of bifacial APTSCs accounted for an increased proportion of the total cost compared with the Cu electrodes^126^, the extended durability and power gain can cover the shortage under actual application scenario.

### Challenge of improving light utilization for APTSCs

Compared with single-junction PSCs, APTSCs feature more absorber layers and divide the solar spectrum across multiple sub-cells. This segmentation offers intrinsic advantages in photon path lengthening and light capture. However, it also introduces new challenges for carrier dynamics engineering. Specifically, stronger interfacial recombination in WBG perovskites—especially in three-junction APTSCs—and higher defect densities in NBG perovskites require novel passivation strategies beyond those used in single-junction devices. From a practical standpoint, increasing the thickness of the NBG perovskite layer can enhance NIR light absorption beyond 950 nm, compensating for its lower absorption coefficient. However, a thicker NBG layer can also worsen defect-induced recombination and hinder charge extraction. This trade-off underscores the need for a coordinated optimization of bulk crystallinity, interfacial passivation, and charge transport layers. Such comprehensive engineering is essential to fully harvest photons across the solar spectrum in APTSCs.

Another roadmap of improving light utilization for APTSCs is integrating tailored surface texturing with engineered light-trapping configurations, which emerge as an effective strategy to suppress reflection losses and minimize angular-dependent optical losses^127^. However, the construction of efficient texturing in APTSCs still faces multiple challenges. Firstly, the solution processing limits the application of traditional etching process and the maintenance of texturing-surface, and it is urgent to develop low temperature and non-destructive texturing-preparation techniques. Secondly, the interface morphology between WBG and NBG perovskite sub-cells in APTSCs may lead to the recombination loss. In addition, complex photon path designs need to be aligned with carrier transport dynamics to prevent electrical performance degradation caused by structural disorder. Future research may focus on self-assembled, nano-scale or hierarchical texturing. Efficient textured design will become a new trend following improved photon capture and offer advantages for the practical deployment of APTSCs and modules. As light management strategies evolving, they not only extend the limits of theoretical performance but also facilitate for scalable and practical implementations of APTSCs.

## Conclusion and outlook

**Fig. 12:**
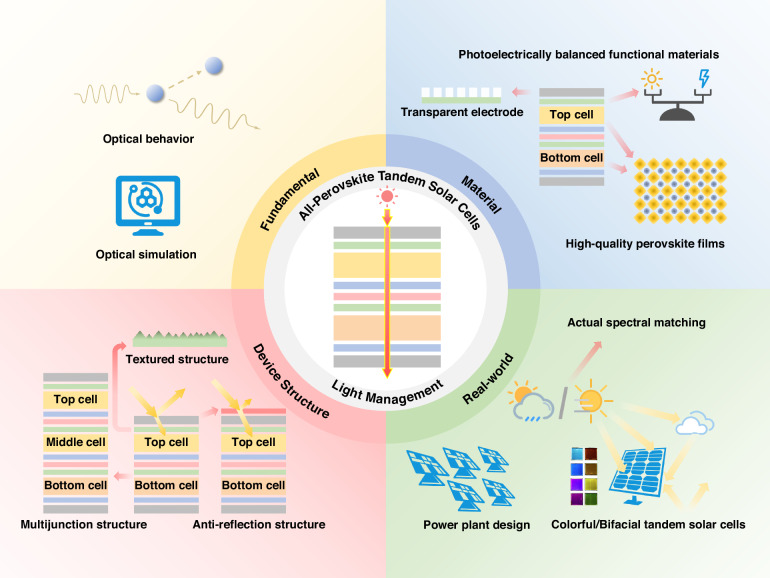
Emerging trends and novel approaches of APTSCs

In recent years, substantial advancements have been achieved in the realm of APTSCs through the dedicated efforts of researchers worldwide. This review highlights breakthroughs in the optical design of APTSCs aimed at maximizing energy utilization and improving PCE. Key strategies include reducing current and optical loss, enhancing light trapping, and achieving comprehensive light utilization. Additionally, the progress of APTSCs with various structural designs has been discussed, demonstrating their potential for practical applications. Nevertheless, there remain numerous opportunities for the future development of APTSCs, as shown in Fig. 12.

To begin with, advancing optical modeling in APTSCs requires a transition from isolated parameter tuning to co-design frameworks based on multi-physical field coupling. This shift involves systematically decoupling the complex interactions among photonic behavior, electric field distribution, and charge transport dynamics. By continuously refining the understanding of electrical loss mechanisms and performing rigorous multi-physics simulations—such as opto-electro-thermal coupling—both optical and carrier transport processes can be more clearly understood. This approach enables a stepwise alignment between theoretical predictions and experimental results, offering guidance for optimizing photon distribution and absorption pathways. Moreover, the optical model should incorporate light scattering effects caused by microstructural inhomogeneities in functional layers, electrode interfaces, and perovskite grain boundaries. Such multidimensional analysis is essential for designing optical structures capability to reduce reflection and harvest photons over a broad angular range.

From a materials-design perspective, APTSCs face a dilemma: optically or electrically. For NBG sub-cells, achieving efficient harvesting of long-wavelength photons requires precise control of crystallization kinetics (e.g., balancing nucleation rate and grain growth competition) to fabricate thick films with large-grained microstructures. Concurrently, WBG sub-cells must maintain exceptional charge transport properties, including high carrier mobility and low trap-state density, while implementing dynamic photon management strategies (bandgap and thickness) to optimize current matching between sub-cells. The electrode architecture also presents dual challenges: front transparent conductive electrodes demand breakthrough solutions to overcome the inherent trade-off between optical transmittance and sheet resistance, while rear electrodes require integrated micro- and nano-structures to simultaneously enhance long-wavelength reflectance and lateral carrier transport efficiency. Functional materials selection now prioritizes electrical compatibility, often sacrificing optical properties. Future efforts should establish quantitative matching models for optical constants (n, k) and implement multiscale optical coupling simulations to comprehensively analyze light-field distribution in multilayer stacks, thereby providing robust physical criteria for advanced material selection.

Moreover, the optical structure design of APTSCs must be synergistically developed with material engineering to optimize device performance. Implementing surface array configurations on glass/air interfaces combined with graded refractive index layers can substantially suppress Fresnel reflection losses while enhancing the harvesting efficiency of broad-spectrum photons. Although micro/nanostructures enable low-loss and wide-angle light trapping across broad spectral ranges, significant challenges persist in controllably constructing textured architectures within functional or absorption layers of APTSCs without compromising electrical properties. This necessitates urgent investigation into planar-compatible texture preservation techniques or post-deposition morphology reconstruction strategies, which are critical for both optical optimization and scalable manufacturing. Furthermore, while high-efficiency 3J-APTSCs theoretically mitigate high-energy photon losses and improve low-energy photon utilization, practical implementation faces obstacles including phase segregation in top sub-cells, intricate current-matching requirements across sub-cells, and precision-demanding ICL designs that collectively hinder performance realization. Addressing these interdependent optoelectronic challenges requires coordinated advancements in multi-scale structural engineering and materials innovation.

Lastly, the spectral conditions encountered in real-world environments should be taken into account for the development of APTSC. In contrast to the reference AM1.5 G spectrum, real-world solar spectral irradiance demonstrates significant geographic variations characterized by distinct atmospheric parameters, particularly aerosol optical depth and precipitable water vapor content. In regions with predominantly clear skies and abundant sunlight, the solar spectrum remains relatively stable with higher overall irradiance. In contrast, areas frequently affected by cloud cover and precipitation experience spectral modifications due to increased scattering and absorption, particularly in the short-wavelength range. These regional spectral variations significantly influence the performance of APTSCs. By optimizing the bandgaps of the WBG and NBG sub-cells to align with the local solar spectrum, APTSCs can achieve a higher PCE in specific deployment environments, demonstrating superior adaptability compared to single-junction PSCs. Fortunately, the real-world solar spectrum is not limited to the standardized spectra used in laboratory settings and can be tailored as needed. The dual-layer light-absorbing structure of APTSCs makes them highly adaptable for applications such as building-integrated photovoltaics (BIPV) and bifacial systems, supporting more efficient deployment of solar power installations.

### Spectral modification

In most cases, the power conversion capability of the APTSCs under discussion is evaluated under the standard of 1-Sun spectrum. However, to achieve a more general current-matching condition, it is possible to artificially modify the solar spectrum by transporting or supplementing the spectrum by up-down conversion materials and the luminous materials.

#### Up-conversion and down-conversion materials

**Fig. 13:**
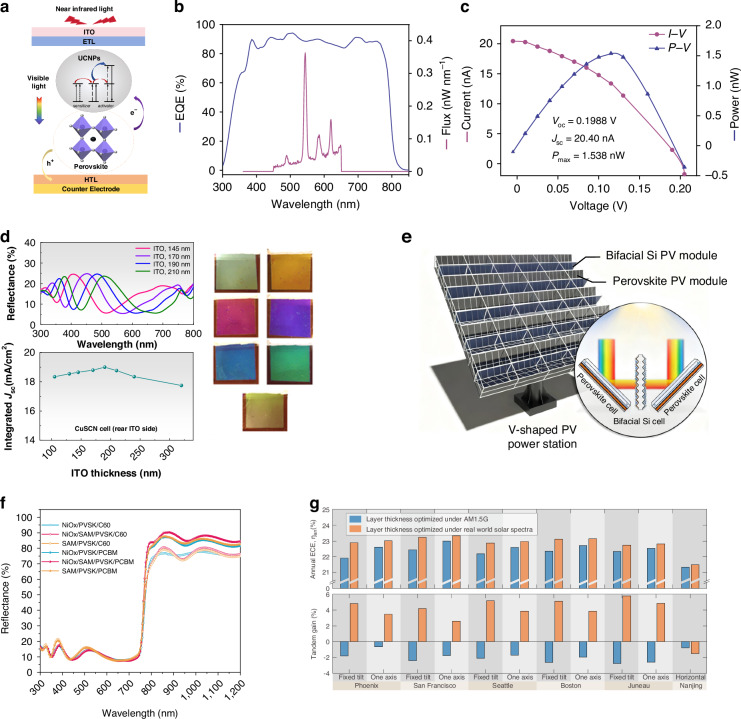
Outlook for APTSCs. **a** Schematically representation of PSCs structure with the UC and DC materials. **b** Spectral responsivity of a Cs_0.05_MA_0.1_FA_0.85_PbI_3_ photovoltaic device and the auto-luminescence spectrum of the TbMel:1%Am sample. **c** *I*–*V* and *P*–*V* curves of the radio-photovoltaic nuclear battery. **d** The reflectance spectra and photographs of PSCs with various ITO electrode thickness. **e** Schematic diagram of a V-shaped perovskite/silicon tandem PV power station. **f** Total reflectance spectra of PSCs with different HTLs and ETLs under light incident from the glass side at 45°. **g** Performance (annual energy conversion efficiency, ECE & annual tandem gain of energy yield against equivalent single-junction) comparison of tandem solar cells optimized under AM 1.5 G and under annual real-world solar spectra. Panels **b** and **c** are reprinted from ref. ^140^ with permission from Springer Nature. Panel **d** is reprinted from ref. ^141^ with permission from American Chemical Society. Panels **e** and **f** are reprinted from ref. ^144^ with permission from Royal Society Chemistry. Panel **g** is reprinted from ref. ^28^ with permission from Elsevier

To leverage the light capturing ability and achieve a balanced photon between two sub-cells, up-conversion (UC) and down-conversion (DC) materials can be used to expand the range of wavelengths absorbed for energy conversion, thus achieving efficient spectrum utilization for APTSCs. UC and DC are defined as converting low-energy or high-energy photons into visible light, further absorbed by perovskite films to generate extra photocurrent^128^. Energy loss for the sub-bandgap photon transmission in the NIR spectral parts of sunlight limits the efficiency of solar cells^129^. UC materials (like NaYF_4_ and CsPbF_3_ et al., doped with the lanthanide elements) can absorb NIR light and up-convert it to higher visible energy photon^130,131^. These UC nanoparticles can be incorporated in CTLs and perovskite layers to improve the light utilization ratio of PSCs^132–135^. However, above UC nanoparticles usually act as severe recombination centers due to the direct contact with the perovskite materials^136^. To circumvent nonradiative recombination, UC materials can be doped in fluorotellurite glass substrate on the front side of PSCs, or placed on the backside of PSCs^137,138^. Moreover, modifying the front conductive substrates with DC materials, such as ZnB_2_O_4_:Mn^2+^ and Ba_9_Sc_2_Si_6_O_24_:Eu^2+^ et al., can effectively convert the UV light into visible light, resulting in higher efficiency and UV stability for the PSCs(Fig. 13a)^139^.

#### Radio-photovoltaic micronuclear battery

Wang et al. proposed a micronuclear battery architecture by integrating a coalescent energy transducer with a perovskite thin-film photovoltaic cell^140^. The coalescence energy sensor was able to achieve a specific self-luminescence spectrum (red line in Fig. 13b), and the PSCs achieved a PCE of 0.889% in this luminescence spectrum region (Fig. 13c). Notably, the cell parameters remained almost unchanged during the tested time of 200 h, indicating the remarkable performance stability of this new type of radio-photovoltaic battery. In the future, this new radio-photovoltaic cell technology could be employed to output photons with specific energy to the current-mismatching 2J-APTSCs, compensating for the current-limited sub-cell to avoid the current-mismatching loss.

### Colorful APTSCs for building-integrated photovoltaics

Colorful PSCs impart improved esthetic quality to design for BIPV. Excellent color tunability across the entire visible spectrum can be achieved by varying the bandgap of perovskite, and the thickness of all functional layers of semitransparent PSCs, resulting from the reflection of the device to a specific spectrum (Fig. 13d)^119,141,142^. In all-perovskite tandem cases, the light absorption ranges from 300 nm to 1100 nm. However, their color tuning range is relatively limited, extending only from purple to yellow by varying the thickness of the transparent ITO rear electrode and the ETL of back sub-cells^141^. Despite this constraint, the ability to produce vivid colors in PSCs presents significant opportunities for esthetic and functional integration in architectural designs.

### All-perovskite tandem solar modules

Research conducted by Boccard et al. showed that series resistance had a slighter impact on the FF values of tandem devices than single-junction devices^143^. It can be attributed to the inherent lower photocurrent density in APTSCs, resulting in reduced dependence on conductivity, thus diminishing the influence of series resistance on FF. This means there is room for efficiency improvement by optimizing the series configuration of all-perovskite tandem solar modules (APTSMs). One strategy is to reduce the thickness of front electrode to minimize the parasitic absorption. Another strategy is to decrease the number of sub-cells in series, thereby reducing the death area and enhancing the overall light utilization of APTSMs. Additionally, the current loss due to the same death area is less pronounced in APTSMs with lower current density compared to single-junction modules, even more inconspicuous in 3J-APTSMs. These factors collectively indicate that APTSMs possess unique optical advantages over single-junction modules in light management and herald a promising future for the advancement of photovoltaic power generation technology.

The operation of APTSMs under real-world solar spectrum is a critical parameter that necessitates thorough investigation prior to the mass production of APTSMs. One primary consideration pertains to the choice between the two configurations: 4T-APTSMs or 2T-APTSMs?

#### Design for 4T-APTSMs and power station

The 4T-APTSM offers significant structural advantages due to its independence from current-matching considerations within each sub-cell. This flexibility allows for innovative designs that can efficiently respond to variations in the solar spectrum without compromising performance. In practical installations, the 4T-configuration can fully exploit the structural advantages, allowing for a versatile design of the module connections. Zheng et al. proposed a creative V-stacked power station, which was installed by multiple V-shaped units in adjacent arrays (shown in Fig. 13e)^144^. In this configuration, the perovskite solar modules on both sides could reflect obliquely incident light to the adjacent bifacial silicon solar module. The perovskite solar module adopted an adaptive light management strategy, introducing NiO_x_/SAM as HTLs, solution-processed PCBM as ETLs and smoothing the reflective silver back electrode, to match the oblique incident light and suppress the diffuse reflection as well as parasitic absorption (Fig. 13f). These enabled a PCE of 27.6% for a V-shaped tandem cell, providing valuable insights for the construction of 4T-APTSM power station.

#### 2T-APTSMs and real-world solar spectra

The 2T-configuration APTSMs are considerably more complex than 4T-configuration under real-world measurement conditions. Achieving current-matching condition within each sub-cell requires considerations of various factors, including temperature, wind speed, angle of incidence, solar spectrum, etc. The research conducted by Gao et al. showed an improvement in the tandem gain of annual energy yield when compared with equivalent single-junction solar cells, with the best results ranging from a 2.8 to a 5.8% increase (Fig. 13g)^28^. This suggested that APTSCs with marginal current-mismatch conditions could even achieve energy gain when operating under real-world and time-varying solar spectra (such as from sunrise to sunset) of specific regions. Furthermore, results also indicated that the power losses due to current-mismatch could be compensated by the extra electricity generated through the tandem structure, relative to single-junction solar cells without such losses. This highlights the necessity of adapting the optical design of APTSMs to local environmental conditions, according to the design principles mentioned in Section “Optical design principle for APTSCs”, to adequately enhance production.

In summary, 4T-APTSMs will be easier and faster to deploy in the short term because they are more flexible in design, easier to produce, and less dependent on solar spectral variations. However, in the long run, 2T-APTSMs have greater market deployment potential due to their lower production cost (less electrodes and packaging materials) and higher power output. From an application perspective, 4T-APTSMs are well-suited for ground power stations that require frequent maintenance and regions with highly variable climatic conditions. In contrast, the more compact 2T-APTSMs are better suited for scenarios prioritizing high power generation efficiency or integration into buildings and curved surfaces. Both architectures are expected to co-exist for an extended period, forming a complementary deployment landscape.

Taking everything into account, compared to single-junction perovskite solar cells and other tandem devices, APTSCs, with later inception, have achieved remarkable progress in a relatively short timeframe. Given the arguments above, it can be noticed that light management in APTSCs is primarily to increase the *J*_sc_ through improved absorption capabilities. The innovative optical design strategies aimed at optimizing performance will undoubtedly play a vital role in the future application of APTSCs. We remain optimistic about the potential for efficiency breakthroughs and the mass production and application potential in other fields of perovskite^145^, anticipating that continued research and development will lead to significant advancements in these technologies, making it a competitive player in the solar energy landscape.
